# Chemical Characterization and Effects of Aqueous *Uncaria tomentosa* Stem-Bark Extract on Keratinocyte Scratch Closure and Inflammatory and Redox Responses

**DOI:** 10.3390/nu18213715

**Published:** 2026-11-09

**Authors:** Euler Ribeiro-Filho, Verônica Farina Azzolin, Vitória Farina Azzolin, Ednea Aguiar Maia-Ribeiro, Suziani da Cruz, Madson do Nascimento, João Batista Xavier Moraes, Josiel do Nascimento Amazonas, Alan Diego da Conceição Santos, Maria Eduarda Chelotti, Débora Filipetto Pulcinelli, Fernanda Barbisan, Ivana Beatrice Mânica da Cruz

**Affiliations:** 1 Graduate Program in Basic and Applied Immunology, Federal University of Amazonas, Manaus 69077-000, AM, Brazil; eulerfilho@gmail.com; 2 GERONTEC Research Center, Open University Foundation of the Third Age, Manaus 69029-040, AM, Brazil; veronica.azzolin@funati.am.gov.br (V.F.A.); vitoriaazzolin@hotmail.com (V.F.A.); edneaamribeiro@hotmail.com (E.A.M.-R.); 3 Amazonas Institute of Forensic Science, Manaus 69090-000, AM, Brazil; suuzycruz@gmail.com (S.d.C.); madsonsn@gmail.com (M.d.N.); fernanda-barbisan.1@ufsm.br (F.B.); 4 Research Group in Sustainable Chemistry and Metabolomics, Federal University of Santa Maria, Santa Maria 97105-900, RS, Brazil; joao.moraes@acad.ufsm.br (J.B.X.M.); alan.santos@ufsm.br (A.D.d.C.S.); 5 Institute of Chemistry, University of Campinas, Campinas 13083-862, SP, Brazil; j272416@dac.unicamp.br; 6 Graduate Program in Pharmacology, Federal University of Santa Maria, Santa Maria 97105-900, RS, Brazil; maria.chelotti@acad.ufsm.br (M.E.C.); deborafpulcinelli@gmail.com (D.F.P.); 7 Biogenomics Laboratory, Health Sciences Center, Federal University of Santa Maria, Santa Maria 97105-900, RS, Brazil; 8 Graduate Program in Gerontology, Federal University of Santa Maria, Santa Maria 97105-900, RS, Brazil

**Keywords:** cat’s claw, skin aging, epidermal repair, HaCaT cells, herbal preparations, inflammaging, metabolomics, phytochemicals, redox homeostasis, scratch assay

## Abstract

Background: Plant-derived bioactive compounds are increasingly investigated as modulators of cellular processes associated with healthy aging. *Uncaria tomentosa* is traditionally consumed as a botanical preparation, but the effects of its aqueous stem-bark extract on keratinocyte repair-related responses remain insufficiently characterized. Methods: The extract was examined using colorimetric assays, DPPH radical scavenging, and untargeted LC-HRMS/MS with molecular networking and in silico class prediction. Cell viability, membrane integrity, extracellular NDUFS7, superoxide, nitric oxide-related products, and cell-cycle distribution were assessed at 1–100 µg/mL. Scratch closure, cytomorphology, IL-1β, and TNF-α were evaluated at 1, 3, and 10 µg/mL. Results: The aqueous extract contained phenolic compounds, flavonoids, tannins, and alkaloids and exhibited DPPH-scavenging activity. LC-HRMS/MS supported a diverse profile including flavonoid glycosides, caffeoylquinic acids, procyanidins, iridoids, and putative triterpenoid glycosides. The extract preserved viability and membrane integrity and did not produce marked basal redox imbalance or increased extracellular NDUFS7. It moderately redistributed cell-cycle phases without a classical arrest pattern. Scratch closure increased at 6 and 24 h. IL-1β decreased at 1 and 3 µg/mL, whereas TNF-α remained broadly stable. Conclusions: The aqueous extract enhanced keratinocyte monolayer closure and selectively modulated inflammatory and redox-metabolic responses relevant to inflammaging. Cellular senescence and the senescence-associated secretory phenotype were not directly demonstrated.

## 1. Introduction

Population aging is accompanied by progressive loss of tissue resilience and slower recovery from injury. In skin, age-associated changes in epidermal proliferation, keratinocyte migration, mitochondrial function, redox homeostasis, extracellular-matrix organization, and immune signaling can delay re-epithelialization and favor chronic inflammation [1,2,3,4].

Cellular senescence contributes to this altered tissue environment. Senescent cells undergo stable proliferative arrest and may develop a senescence-associated secretory phenotype (SASP) that includes cytokines, chemokines, growth factors, and matrix-remodeling enzymes [5,6,7]. The effects of senescence during repair are context-dependent: transient senescent-cell signaling can support normal wound healing, whereas persistent senescent-cell accumulation and chronic SASP activity can impair regeneration [8].

Plant foods and traditionally consumed botanical preparations contain numerous nonessential bioactive compounds capable of affecting inflammatory and redox signaling. Such compounds should be distinguished from essential nutrients, but their biological activities are relevant to nutritional geroscience and healthy-aging research [9]. Flavonoids, other polyphenols, and alkaloids are of particular interest because they can interact with pathways involved in oxidative stress, NF-κB signaling, mitochondrial homeostasis, and cellular senescence [10]. Nevertheless, direct exposure of cultured cells to a botanical extract does not establish bioavailability or efficacy after oral consumption; it instead provides mechanistic evidence that requires subsequent pharmacokinetic and in vivo validation.

*Uncaria tomentosa* (Willd. ex Schult.) DC. (Rubiaceae), commonly known as cat’s claw, is an Amazonian medicinal plant used in South American traditional medicine and included in Brazil’s National List of Medicinal Plants of Interest to the Unified Health System. Decoctions and other preparations of its stem bark have been used in contexts involving inflammation and immune-related disorders [11,12]. Its phytochemical composition includes oxindole alkaloids as well as polyphenols, flavonoids, proanthocyanidins, quinic-acid derivatives, iridoids, and triterpenoids [12,13].

Ethnomedicinal reports describe the traditional use of *Uncaria tomentosa* for wound care in Peru [11,14]. An ethnobotanical survey in northern Peru specifically documented aqueous preparations of leaves and stems for topical application, including poultices and the application of soaked leaves to wounds [14].

However, a botanical screening study reported reduced scratch closure in HaCaT keratinocytes exposed to *U. tomentosa* bark extracts prepared with 20% or 70% ethanol, without assessing accompanying inflammatory and redox responses [15].

These responses warrant investigation because re-epithelialization involves the coordinated migration and proliferation of keratinocytes to restore epidermal integrity [2]. Research on the molecular regulation of keratinocyte progenitors has shown that disruption of their transition from inflammatory to proliferative and migratory states delays re-epithelialization [16]. Metabolic disturbances can also impair keratinocyte function. For example, exposure to high glucose reduces cultured keratinocyte migration and, after prolonged exposure, proliferation, alongside changes in matrix metalloproteinase activity and focal adhesion signaling [17].

Together, these findings support evaluating keratinocyte closure alongside inflammatory and redox-related endpoints. However, the effects of an aqueous *U. tomentosa* stem-bark extract on these complementary responses remain insufficiently characterized. Therefore, the present study combined chemical characterization of an aqueous extract with assessments of HaCaT scratch closure, cell viability, membrane integrity, cell-cycle distribution, inflammatory cytokines, and redox-related endpoints.

## 2. Materials and Methods

### 2.1. Reagents and Instruments

All reagents and solvents were of analytical and LC-HRMS/MS grade and were purchased from Sigma-Aldrich^®^ (Saint Louis, MO, USA). All aqueous solutions were prepared using purified water obtained from a Milli-Q^®^ IQ 7000 ultrapure water purification system (Merck, Darmstadt, Hesse, Germany). Cell culture media and reagents were obtained from Invitrogen Life Technologies^®^ (Karlsruhe, Baden-Württemberg, Germany). Flow cytometry analyses were performed using an Attune™ NxT Acoustic Focusing Cytometer (Thermo Fisher Scientific, Waltham, MA, USA). Chemicals used for flow cytometry analyses were purchased from BD Pharmingen (Broomfield, CO, USA).

Molecular assays were performed using reagents from Ludwig Biotec^®^ (Alvorada, Rio Grande do Sul, Brazil), Invitrogen Life Technologies^®^ (Carlsbad, CA, USA), Bio-Rad Laboratories (Hercules, CA, USA), and QIAGEN Biotechnology (Hilden, North Rhine-Westphalia, Germany).

LC-HRMS/MS analysis. The samples were analyzed using a high-performance liquid chromatography (HPLC) system (Shimadzu^®^, Tokyo, Japan), coupled to a quadrupole time-of-flight high-resolution mass spectrometer (Q-TOF-MS; Bruker Daltonics^®^, Fremont, CA, USA). Cells were cultured in incubator Panasonic^®^ (Osaka, Osaka Prefectur, Japan).

Biochemical assays involving spectrophotometric and fluorometric analyses were conducted using a SpectraMax i3 multimode platform (Molecular Devices, San José, CA, USA). A Thermo Scientific NanoDrop™ 1000 spectrophotometer (Waltham, MA, USA) was used for molecular analyses. Conventional thermocyclers (Corning Inc., Somerville, MA, USA) and a Rotor-Gene Q 5plex HRM system (QIAGEN Biotechnology, Hilden, North Rhine-Westphalia, Germany) were also employed.

### 2.2. Plant Material and Preparation of the Aqueous Extract

Commercially packaged *U. tomentosa* stem bark was obtained from Dermapelle Compounding Pharmacy (Santa Maria, Rio Grande do Sul, Brazil; manufacturing batch: AUTO23051; manufacturing date: 9 April 2025; expiration date: 8 December 2028). Powdered stem bark (50 g) was combined with distilled water (500 mL) and maintained under continuous agitation for 8 h at 37 °C. The suspension was filtered, and the filtrate was lyophilized for 48 h at −45 °C. The apparent extraction yield was estimated indirectly by gravimetric difference. After extraction and filtration, the insoluble bark residue remaining on the filter was dried and weighed (42.5 g). The apparent extraction yield was calculated as the difference between the initial mass of powdered stem bark and the dried residue mass, divided by the initial mass: apparent extraction yield (%) = [(50 g − 42.5 g) ÷ 50 g] × 100 = 15% (*w*/*w*). The lyophilized filtrate was not weighed to determine this yield; therefore, the 15% value represents an indirect estimate based on mass difference and not a directly measured recovery of the lyophilized aqueous extract. The lyophilized material was aliquoted and stored at 2 °C. Immediately before the biological assays, it was reconstituted in sterile distilled water at the required concentrations.

### 2.3. Colorimetric Phytochemical Assays and DPPH-Scavenging Activity

Total phenolic content was assessed using the Folin–Ciocalteu method [18]. For the analysis, the sample was subjected to reaction with 0.5 mL of Folin–Ciocalteu reagent, 8.5 mL of Na_2_CO_3_ (7.5%), and 12 mL of distilled water. The mixture was incubated for 2 h, after which absorbance was measured at 765 nm. The phenolic content was calculated using a calibration curve prepared with gallic acid (y = 0.4824x + 0.0434; r^2^ = 0.997), and the results are expressed as milligrams of gallic acid equivalents per gram of *U. tomentosa* (mg GAE/g). Flavonoid content was determined using a colorimetric assay based on complex formation with aluminum chloride (AlCl_3_) [19]. The reaction mixture contained methanol, sodium nitrite, AlCl_3_, and sodium hydroxide, with the final volume adjusted using methanol. After 40 min of incubation, absorbance was recorded at 490 nm. Quantification was performed using a calibration curve described by the equation y = 1.7033x + 0.0348 (r^2^ = 0.9918), and the results are expressed as milligrams of quercetin equivalents per gram of extract (mg QE/g).

Tannin content was determined using the vanillin method, following the procedure adapted from [20]. Tannins were extracted using 20% methanol containing 1% HCl, followed by reaction of the resulting extract with a vanillin solution. After 15 min of reaction, absorbance was measured at 490 nm. Quantification was based on a catechin calibration curve (y = 0.0813x + 0.0331; r^2^ = 0.995), and the results are expressed as milligrams of catechin equivalents per gram of extract (mg CE/g). Alkaloid content was determined based on the reaction of Dragendorff’s reagent with the nitrogen atoms present in alkaloids [21]. The results are expressed as milligrams of bismuth nitrate equivalents per gram of extract (mg BNE/g), using a calibration curve (y = 1.3466x − 0.002; r^2^ = 0.9988).

Antioxidant capacity was evaluated using the DPPH (2,2-diphenyl-1-picrylhydrazyl) radical-scavenging assay [22]. Briefly, 100 µL of DPPH solution (200 µM) was mixed with 100 µL of the extract at concentrations ranging from 1 to 1000 µg/mL. The reaction mixtures were incubated for 30 min under light-protected conditions, after which absorbance was measured at 517 nm. The percentage of DPPH radical inhibition was calculated by comparing the absorbance of the samples with that of the DPPH control. Antioxidant activity was evaluated based on the concentration-dependent reduction in DPPH absorbance, and the IC_50_ value, corresponding to the extract concentration required to inhibit 50% of the DPPH radical, was estimated from the concentration–response curve using four-parameter logistic regression in GraphPad Prism. Rutin was used as a positive control, and a calibration curve was prepared using rutin concentrations ranging from 1 to 1000 µg/mL. All measurements were performed in triplicate.

### 2.4. LC-HRMS/MS Analysis

Approximately 5 mg of aqueous extract of *U. tomentosa* was dissolved and analyzed using a high-performance liquid chromatography (HPLC) system (Shimadzu, Tokyo, Japan) equipped with an autosampler maintained at 8 °C and a thermostatically controlled column compartment, and coupled to a quadrupole time-of-flight high-resolution mass spectrometer (Q-TOF-MS) (Bruker Daltonics, Fremont, CA, USA). Chromatographic separation was performed using a reversed-phase Shim-pack XR-ODS II column (100 × 3.0 mm, 2.2 µm particle size). Gradient elution was as follows: 5–100% B solution [ACN (0.1% formic acid)] from 0 to 60 min, 100% B from 60 to 70 min, and 100–5% B from 70 to 80 min. The flow rate was kept at 0.5 mLmin^−1^, and the column temperature at 37 °C. The injection volume was 20.0 µL.

Detections were carried out using a Q-TOF mass spectrometer equipped with an ESI interface, using data-dependent acquisition (DDA) mode. The parameters of the ionization source (negative mode) were as follows: capillary potential of 4.5 kV, nebulizer gas pressure (nitrogen) of 2.0 bar, drying gas flow (nitrogen) of 6 Lmin^−1^, and gas temperature of 250 °C. The acquisition range was from *m*/*z* 100 to 800. The instrument was calibrated with 2.67 mM sodium iodide, allowing accurate mass measurements of each peak from the total ion chromatograms (TICs).

### 2.5. LC-MS/MS Data Processing and Metabolite Annotation

The raw data files (.lcd) were converted to .mzML format using MSConvert (ProteoWizard, Palo Alto, CA, USA) and processed in MZmine 4.10.6 using the built-in Processing Wizard (mzwizard), which provides a standardized and reproducible workflow for LC–QTOF–MS data processing [23]. Processing included chromatogram construction, ADAP deconvolution, isotope and adduct grouping, feature alignment, gap filling, and feature-list generation. The retention-time range was 1.0–80.0 min; at least four consecutive scans were required; the estimated chromatographic peak width was 0.05 min; and the MS^1^ and MS^2^ noise thresholds were 2.0 × 10^3^ and 1.0 × 10^2^, respectively, with a minimum feature height of 1.0 × 10^3^. The mass tolerances were 0.0050 Da (20 ppm) for scan-to-scan comparisons, 0.0015 Da (3 ppm) for within-sample grouping, and 0.0040 Da (8 ppm) for between-sample alignment. MS/MS spectra were exported in Mascot Generic Format (.mgf), containing fragment-ion information associated with the corresponding precursor ions. A feature quantification table was also exported in comma-separated values (.csv) format. The resulting .mgf and .csv files were subsequently used for molecular networking and SIRIUS analyses [24].

Feature-based molecular networking was conducted on the GNPS platform [23]. Precursor and fragment ion tolerances were 0.02 Da. Network edges required a cosine score of at least 0.75 and at least four matched fragment ions; library matches required a cosine score of at least 0.75 and at least three matched peaks. The maximum molecular-family size was 100 nodes, and the 10 most similar neighbors per node were retained. The FBMN job on GNPS can be found at https://gnps2.org/status?task=cd90fd0c11a046efad2132315e0f9e1a (accessed on 24 October 2026). Networks were visualized in the Cytoscape software (version 3.10.4, Cytoscape Consortium, San Diego, CA, USA), where each node corresponds to ion features of specific *m*/*z* ratio and retention time [25,26].

The SIRIUS software (version 6.4.0) was used to explore possible molecular-formula prediction, ZODIAC re-ranking, CSI:FingerID, spectral-library matching, and CANOPUS chemical-class prediction [25,27]. The QTOF mass tolerance for MS/MS was 10 ppm, and formula generation was restricted to H, C, N, O, P, and S. Metabolite annotations were assigned according to Metabolomics Standards Initiative principles [26]. Because authentic standards were not analyzed, annotations were considered putative.

### 2.6. HaCaT Cell Culture and Experimental Design

HaCaT immortalized human keratinocytes (CLS Cell Lines Service GmbH, Cat. No. 300493, Eppelheim, Germany) were maintained in Dulbecco’s modified Eagle medium supplemented with 10% fetal bovine serum and 1% penicillin–streptomycin at 37 °C in a humidified atmosphere containing 5% CO2. The medium was replaced every 2 days, and cells were subcultured at approximately 80–90% confluence using trypsin–EDTA. Cells in exponential growth with adequate viability were used [28]. An initial concentration range of 1–100 µg/mL was used for safety, redox, and cell-cycle assessments. Concentrations of 1, 3, and 10 µg/mL were selected for scratch-closure, cytomorphological, and cytokine analyses.

### 2.7. Cell Viability, Membrane Integrity, and Redox Assays

Metabolic viability was evaluated after 24 h of treatment using the MTT assay. Cells cultured in 96-well plates were washed with phosphate-buffered saline (PBS; 0.01 M, pH 7.4), followed by the addition of 90 µL of PBS and 10 µL of MTT solution (5 mg/mL in PBS). After incubation for 4 h at 37 °C, the supernatant was removed, the formazan crystals were dissolved in 200 µL of dimethyl sulfoxide, and absorbance was measured at 560 nm [29]. Plasma membrane integrity was assessed after 24 h of treatment by propidium iodide exclusion and flow cytometry [30]. Griess-reactive products in the culture medium were measured after 24 h at 540 nm as an indirect indicator of nitric oxide-related metabolism [31]. Superoxide production was estimated after 24 h by measuring the reduction in nitroblue tetrazolium to formazan using a colorimetric assay [32].

### 2.8. Cell-Cycle Analysis

Cell-cycle distribution was determined by PI DNA-content analysis. Cells were seeded in six-well plates at 5 × 10^4^ cells/well in 2 mL, treated for 24 h, harvested, washed with PBS, and fixed in 70% ethanol overnight at −20 °C. Cells were then washed and incubated for 40 min at 37 °C in 500 µL PBS containing 50 µg/mL PI, 0.1 mg/mL RNase A, and 0.05% Triton X-100. DNA-content distributions were acquired by flow cytometry using an Attune NxT Flow Cytometer (Thermo Fisher Scientific), with BL1/BL2 filters, and 10,000 events were acquired per sample. The data were analyzed using Attune Cytometric Software version 7.2 and used to estimate the proportions of cells in G0/G1, S, and G2/M phases.

### 2.9. Scratch-Closure and Cytomorphological Analyses

HaCaT cells were seeded in 96- or 12-well plates and grown to 90–100% confluence. A linear cell-free gap was produced with a sterile 200 µL pipette tip in 96-well plates or a 1000 µL tip in 12-well plates. Detached cells were removed by two washes with culture medium. Images were acquired at 0, 6, and 24 h under standardized magnification, illumination, and exposure conditions, using reference points to follow the same fields. Scratch closure was calculated relative to the open area at 0 h. Because proliferation was not reported as pharmacologically blocked, the outcome is described as monolayer or scratch closure rather than migration alone [33,34].

After 24 h, cultures in 12-well plates were fixed for 15 s and stained for 15 s with a methylene blue-based Panoptic Kit protocol, washed with PBS, air-dried, and imaged by light microscopy. Images were converted to 8-bit grayscale in Fiji/ImageJ (Version 1.54p 17) corrected for background, segmented by automated thresholding, and analyzed using predefined size and circularity filters. Cell area, aspect ratio, roundness, and solidity were used to classify four operational phenotypes: cobblestone keratinocytes, proliferative-appearing keratinocytes, apoptotic-like morphology keratinocytes, and enlarged keratinocytes. These categories were morphology-based and were not treated as molecularly validated cell states.

### 2.10. Immunoassays

NDUFS7, IL-1β, and TNF-α were quantified using commercial 96-well ELISA kits (Elabscience Biotechnology, Houston, TX, USA) according to the manufacturer’s instructions. The reported calibration equations were IL-1β, y = 300.96x − 24.094 (R^2^ = 0.9928); TNF-α, y = 0.0023x + 0.1549 (R^2^ = 0.9673); and NDUFS7, y = 0.0752x + 0.2914 (R^2^ = 0.9582). The standard-curve ranges were 3.9–250 pg/mL for IL-1β, 7.8–500 pg/mL for TNF-α, and 0.312–20 ng/mL for NDUFS7. Cytokines were expressed as pg/mL and NDUFS7 as ng/mL. Values below the analytical range were recorded as below the limit of quantification. NDUFS7 in culture supernatants was treated as an exploratory indicator of release of a mitochondrial complex I-associated protein rather than as a validated stand-alone biomarker of mitochondrial damage.

### 2.11. Statistical Analysis

Data are presented as the mean ± standard deviation (SD) from three independent biological experiments (*n* = 3), each performed in technical triplicate. Technical replicates were averaged within each independent experiment before statistical analysis. Individual wells, microscopic fields, and cells were not considered independent biological replicates. Scratch-closure data obtained from independent wells at 6 and 24 h were analyzed using one-way or two-way analysis of variance (ANOVA) without repeated measures, with treatment and time as factors, followed by Tukey’s or Bonferroni multiple-comparison test. Flow-cytometric, spectrophotometric, immunoassay, and cytomorphological data were analyzed using one-way ANOVA followed by Tukey’s multiple-comparison test. Statistical analyses were performed using GraphPad Prism version 9.5.1. Differences were considered statistically significant at *p* < 0.05. In graphs and tables, groups that do not share the same letter are significantly different.

## 3. Results

### 3.1. Phytochemical Composition and Antioxidant Activity

Colorimetric assays indicated that the aqueous extract contained phenolic compounds, flavonoids, condensed tannins, and alkaloids (Table 1). The DPPH IC50 was 148.5 ± 0.09 µg/mL.

Library matching and fragmentation patterns supported the presence of flavonoid glycosides, proanthocyanidins, hydroxycinnamic/quinic-acid derivatives, iridoid and secoiridoid glycosides, and putative glycosylated pentacyclic triterpenoids. Representative annotations included rutin, quercetin-3-O-glucoside, kaempferol glycosides, afzelin, taxifolin-3-glucoside, procyanidin B1, chlorogenic-acid isomers, isochlorogenic acid C, loganic acid, geniposidic acid, and sweroside, as shown in Table 2. A complete list of the 44 MS-based putative metabolite annotations in the *U. tomentosa* extract is provided in Table S1. For the representative annotations, accurate-mass errors were within approximately ±2 ppm, and several library matches exhibited cosine scores above 0.90.

**Figure 1:**
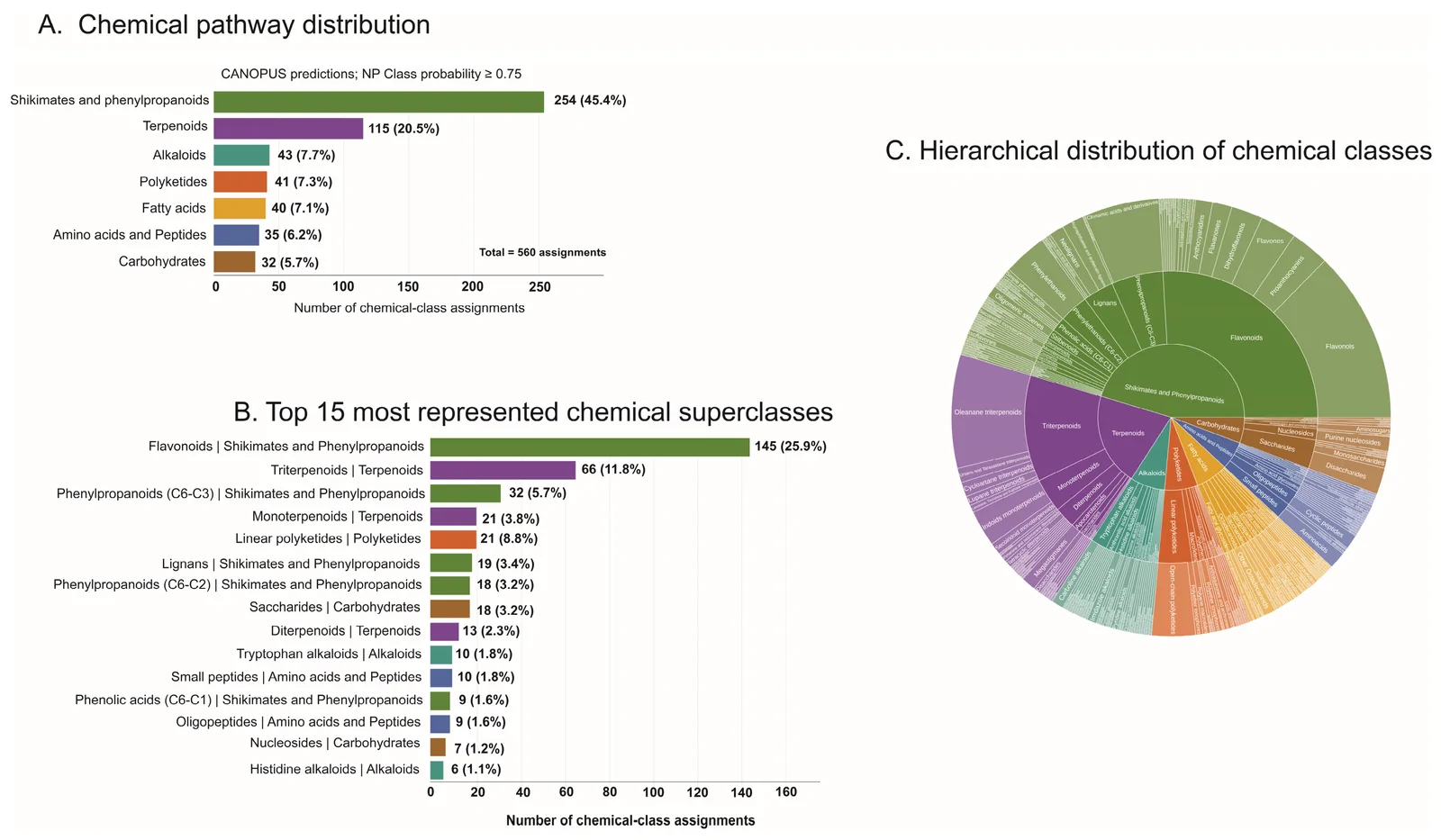
Chemical-class distribution of the aqueous *U. tomentosa* extract based on CANOPUS predictions. (**A**) Pathway-level assignments retained at natural-product class probability ≥ 0.75. (**B**) Hierarchical sunburst of predicted classes. (**C**) Most represented classes and subclasses. These are computational class assignments derived from MS/MS data.

**Figure 2:**
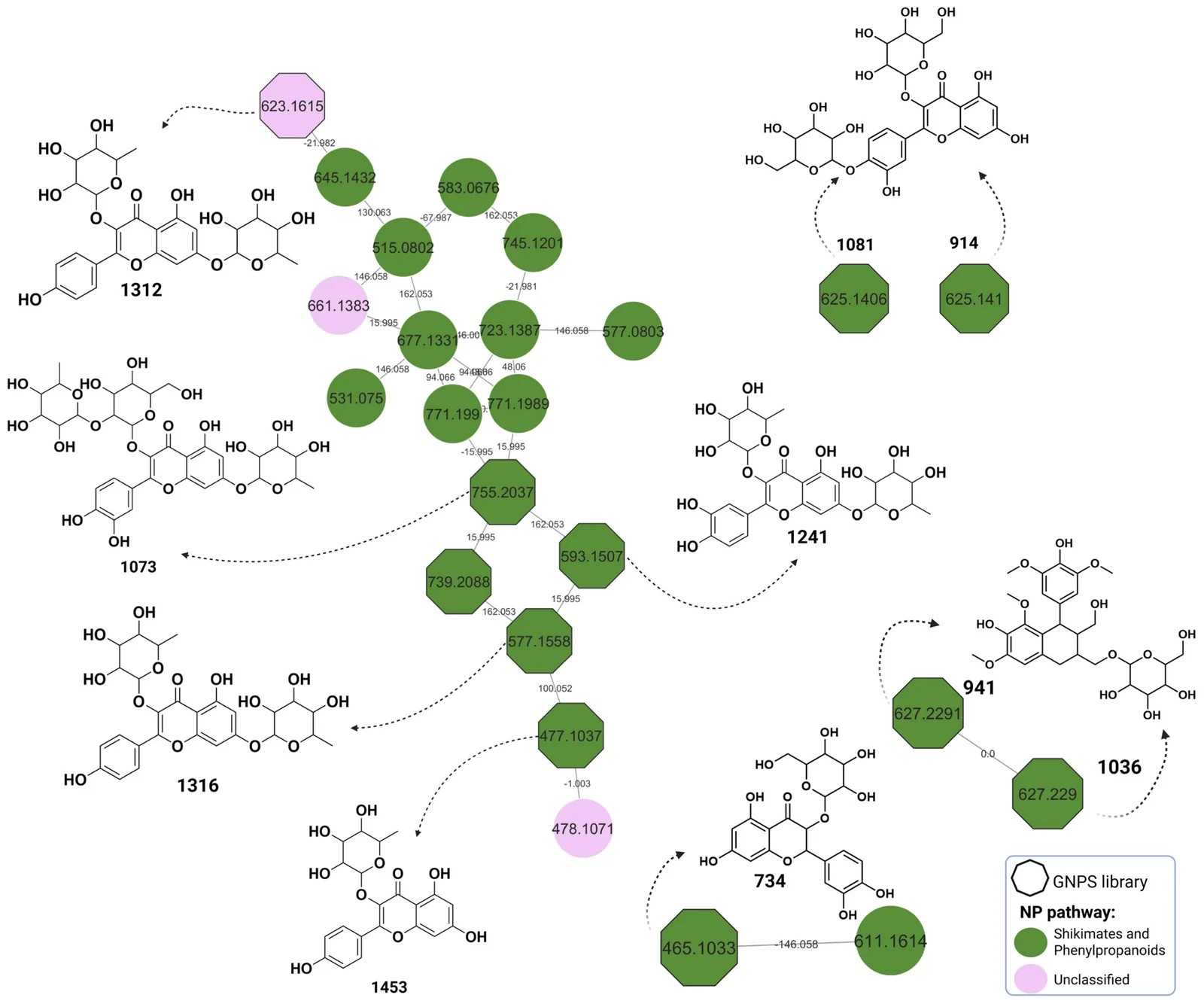
Shikimate/phenylpropanoid-enriched molecular subnetwork from the aqueous *U. tomentosa* extract. Nodes represent MS/MS features, and edges connect features with similar fragmentation patterns. Node colors represent CANOPUS-predicted pathways; octagonal nodes indicate GNPS library matches. Compound structures denote putative annotations.

**Figure 3:**
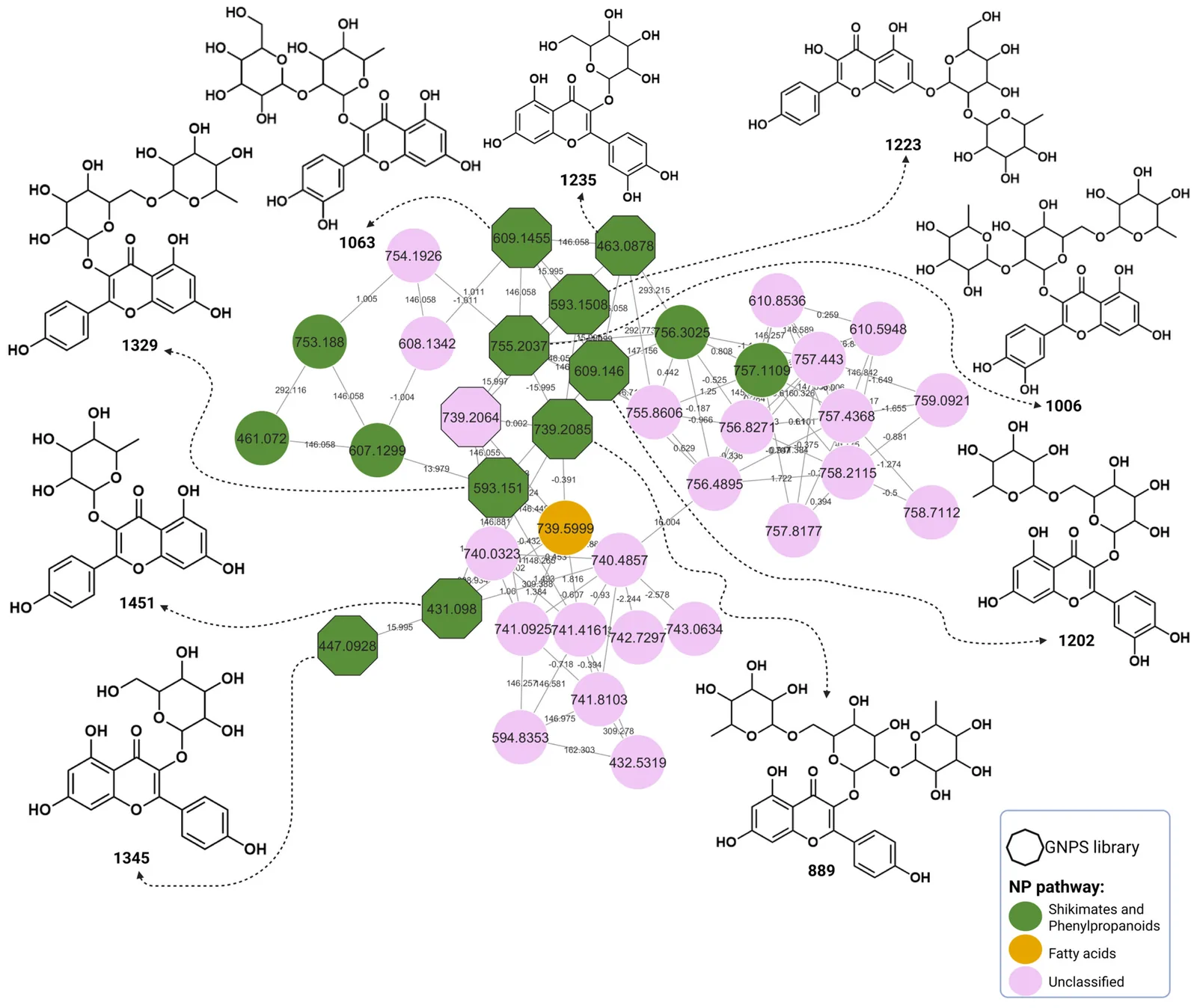
Terpenoid-enriched molecular subnetwork from the aqueous *U. tomentosa* extract. Nodes represent MS/MS features, and edges connect features with similar fragmentation patterns. Node colors represent CANOPUS-predicted pathways; octagonal nodes indicate GNPS library matches. Compound structures denote putative annotations.

**Figure 4:**
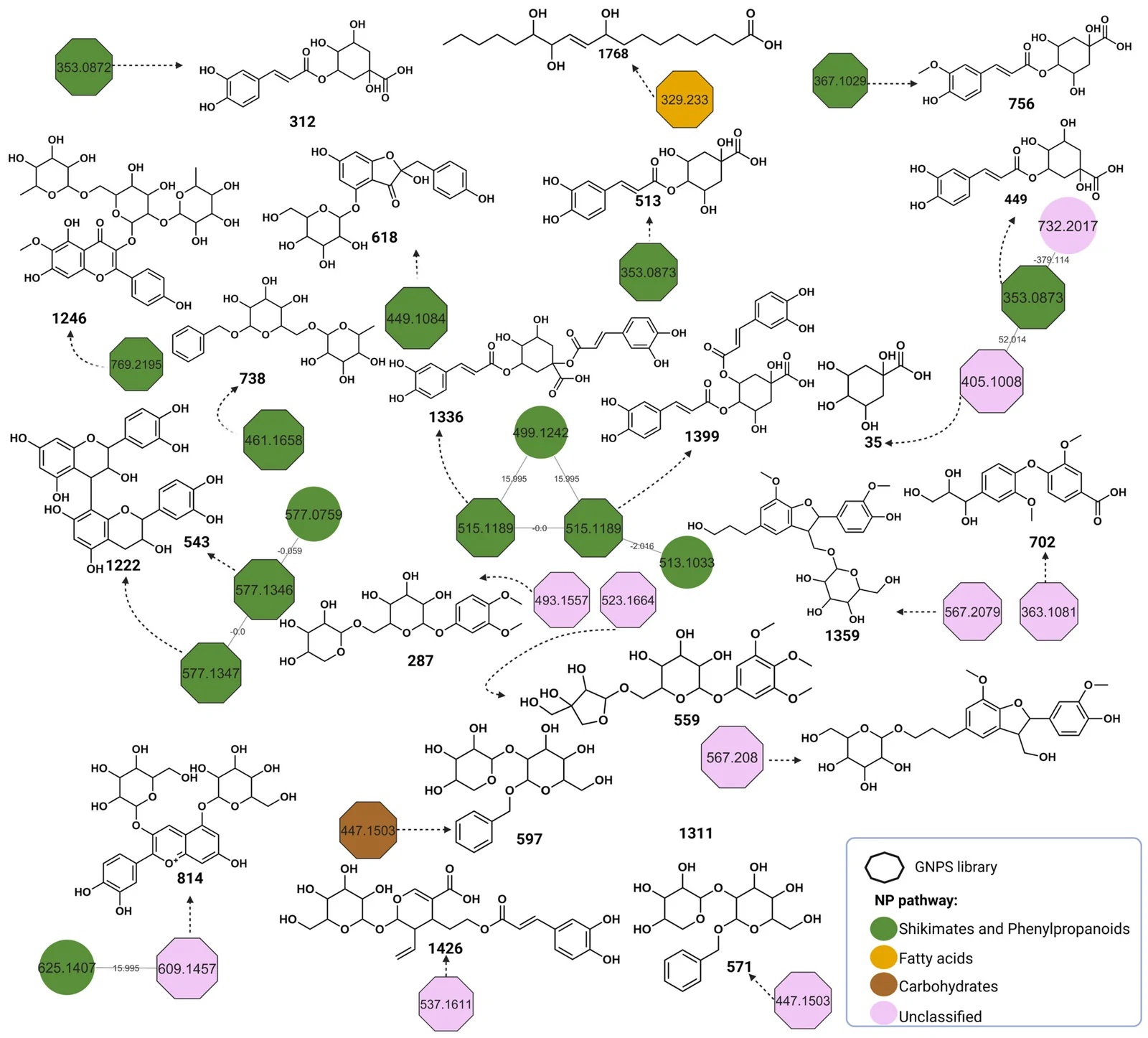
Representative molecular subnetwork showing diverse metabolite families detected in the aqueous extract of *Uncaria tomentosa.* Nodes represent MS/MS features, and edges connect features with similar fragmentation patterns. Node colors represent CANOPUS-predicted pathways; octagonal nodes indicate GNPS library matches. Compound structures denote putative annotations.

The chemical diversity of *U. tomentosa* extract was further examined using the hierarchical class predictions generated by CANOPUS, a deep neural network-based computational tool designed for the systematic classification and annotation of chemical compound classes directly from fragmentation spectra (MS/MS) [25]. Using an NP class probability threshold of ≥0.75, a total of 560 chemical-class assignments were retained. Shikimate/phenylpropanoid-related classes predominated (254 assignments; 45.4%), followed by terpenoids (115; 20.5%), alkaloids (43; 7.7%), polyketides (41; 7.3%), fatty acids (40; 7.1%), amino acids and peptides (35; 6.3%), and carbohydrates (32; 5.7%) (Figure 1). Molecular subnetworks enriched in shikimate/phenylpropanoid-related, terpenoid-related, and other metabolite families are shown in Figure 2, Figure 3 and Figure 4, respectively.

### 3.2. The Aqueous Extract Did Not Cause Overt Cytotoxicity or Marked Basal Redox Imbalance

**Figure 5:**
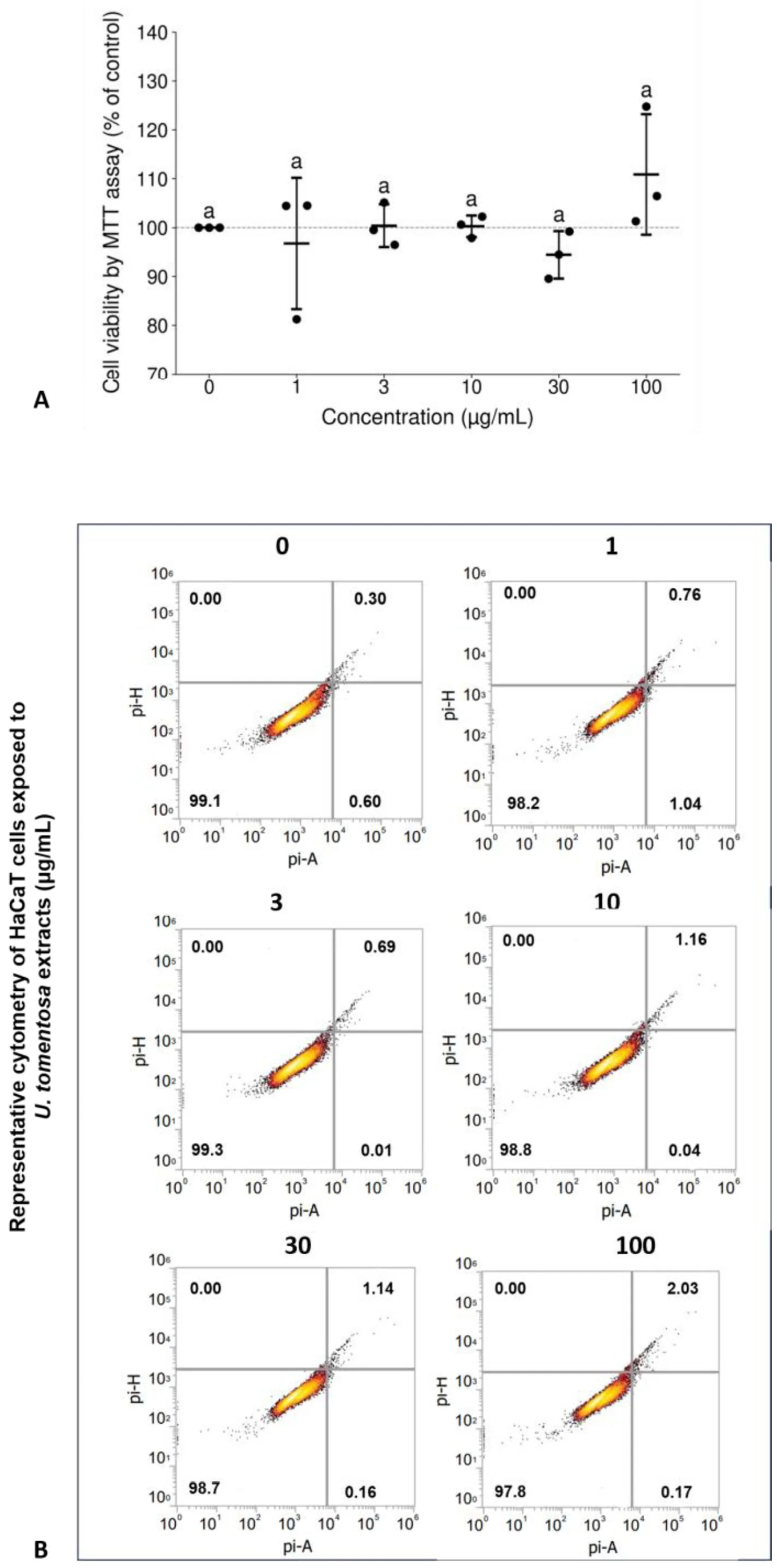
Cytotoxicity-related endpoints in HaCaT keratinocytes exposed to aqueous *U. tomentosa* extract (1–100 µg/mL). (**A**) MTT reduction expressed as a percentage of the untreated control (0 µg/mL). Each point represents the mean of technical replicates from one independent biological experiment (*n* = 3). Horizontal lines and error bars indicate mean ± SD. Groups sharing the same letter do not differ significantly according to Tukey’s multiple-comparison test following one-way ANOVA (α = 0.05). (**B**) Representative propidium iodide (PI) flow cytometry plots for the indicated concentrations (µg/mL). Numbers within quadrants indicate percentages of events.

Across 1–100 µg/mL, MTT reduction remained close to control values after 24 h, and PI exclusion did not reveal a concentration-dependent increase in membrane-compromised cells (Figure 5). Extracellular NDUFS7 levels were not significantly elevated, providing no evidence of treatment-associated release of this mitochondrial complex I subunit under the conditions tested. These assays do not exclude more subtle mitochondrial effects.

**Figure 6:**
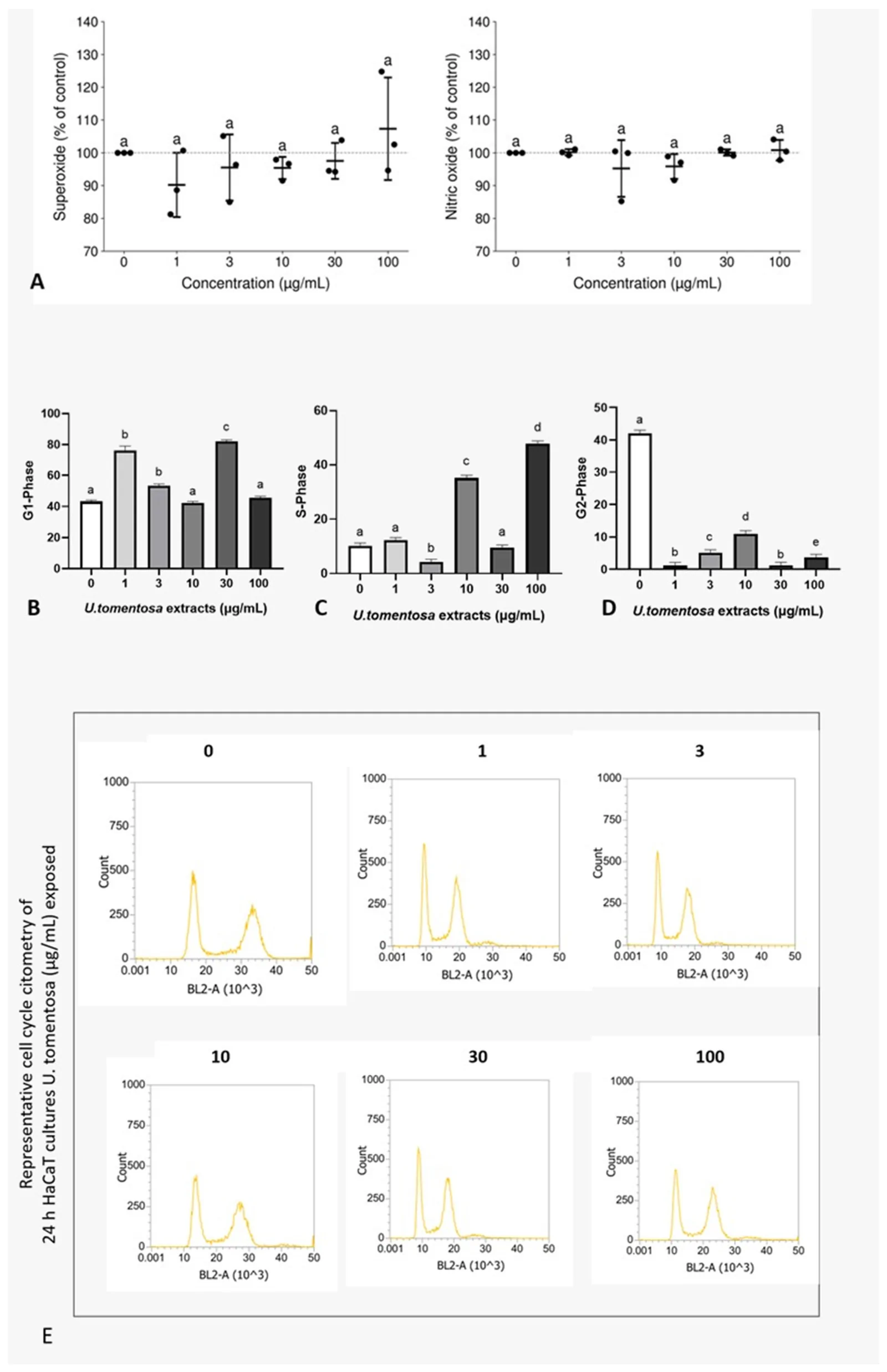
Redox-related markers and cell-cycle distribution in HaCaT keratinocytes exposed to aqueous *U. tomentosa* extract (1–100 µg/mL) for 24 h. (**A**) Superoxide-dependent NBT reduction (**left**) and Griess-reactive products (**right**), expressed as percentages of the untreated control (0 µg/mL). Each point represents the mean of technical replicates from one independent biological experiment (*n* = 3). Horizontal lines and error bars indicate mean ± SD. (**B**–**D**) Percentages of cells in G0/G1, S, and G2/M, respectively, presented as mean ± SD. (**E**) Representative propidium iodide (PI) DNA-content histograms for the indicated extract concentrations (µg/mL). Comparisons among concentrations were performed separately for each endpoint using one-way ANOVA followed by Tukey’s multiple-comparison test. Within each graph, groups sharing the same letter do not differ significantly, whereas different letters indicate *p* < 0.05.

Superoxide-dependent NBT reduction and Griess-reactive products were not markedly increased after 24 h (Figure 6A). Cell-cycle analysis showed moderate redistribution among G0/G1, S, and G2/M phases (Figure 6B–E). The proportions of cells in G0/G1 increased at 1, 3, and 10 µg/mL relative to control, while selected higher concentrations increased the S-phase fraction and most treatments reduced the G2/M fraction. The response was nonmonotonic and did not show pronounced accumulation in a single phase; therefore, it was interpreted as altered short-term cell-cycle distribution rather than evidence of stable senescence-associated arrest.

### 3.3. The Aqueous Extract Enhanced Keratinocyte Monolayer Closure

**Figure 7:**
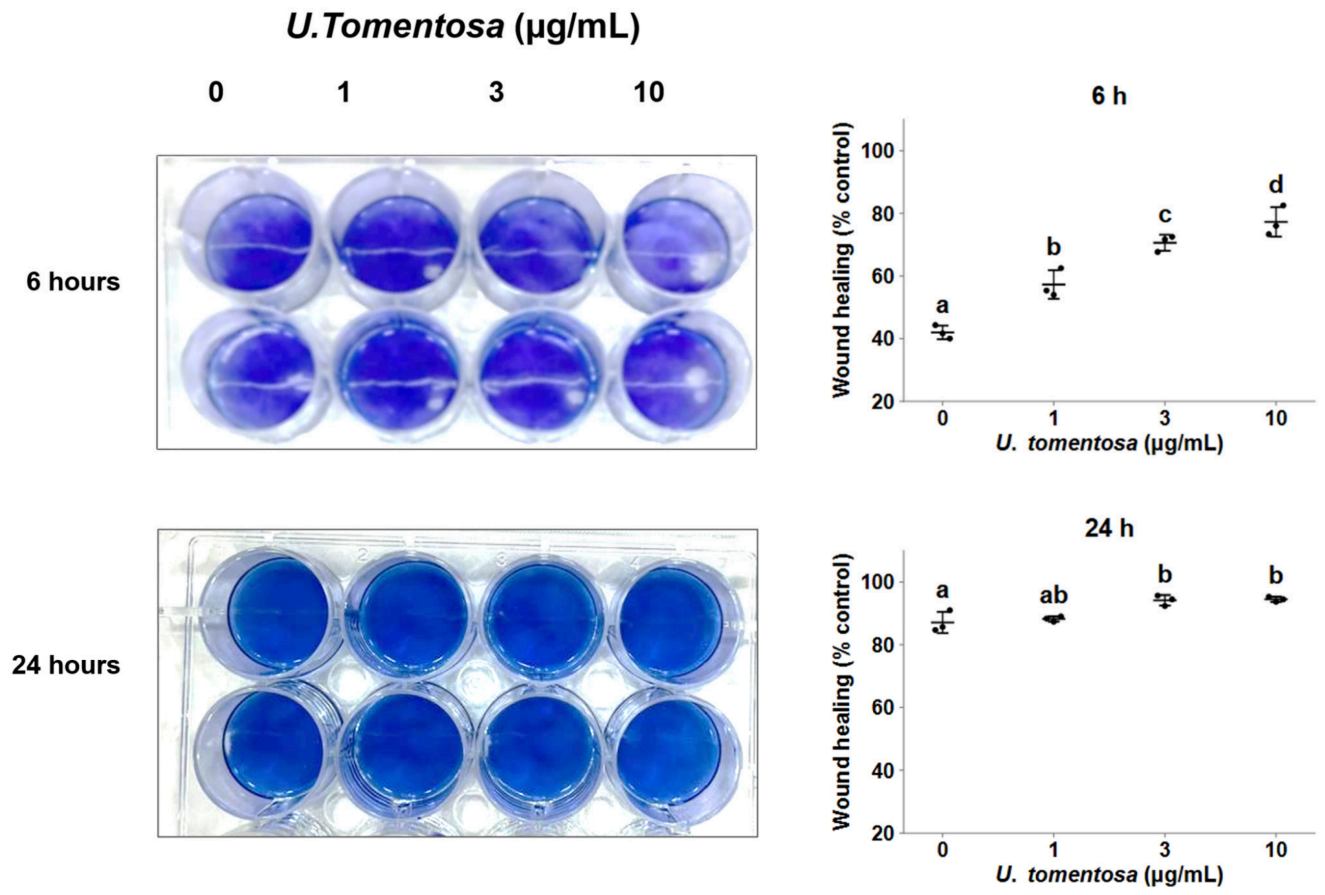
Scratch assay in HaCaT keratinocyte monolayers treated with aqueous *U. tomentosa* extract (0, 1, 3, and 10 µg/mL). Representative photographs show fixed and stained cultures at 0, 6, and 24 h, arranged from top to bottom. At 0 h, only untreated control cultures are shown. At 6 and 24 h, columns correspond to increasing extract concentrations from left to right, with duplicate wells arranged vertically. Separate cultures were fixed and stained at each time point; therefore, the photographs do not represent sequential imaging of the same wells. The graph shows the estimated percentage of scratch closure at 6 and 24 h, calculated relative to the initial cell-free area of independent untreated control cultures at 0 h. Data are presented as mean ± SD from three independent experiments, each summarized from three technical measurements. The Shapiro–Wilk test did not detect a significant departure from normality in the within-experiment model residuals (W = 0.9773; *p* = 0.8421). Different letters indicate statistically significant differences among groups (*p* ≤ 0.05), as determined by two-way ANOVA followed by Tukey’s multiple-comparison test.

Scratch closure increased over time in all groups, reflecting the intrinsic repair capacity of confluent HaCaT cultures. At 6 h, each tested concentration (1, 3, and 10 µg/mL) increased closure relative to the untreated control, with the largest response at 10 µg/mL. At 24 h, closure remained greater in the 3 and 10 µg/mL groups than in the control (Figure 7). Because proliferation was not reported as blocked, these findings demonstrate enhanced monolayer closure but do not isolate migration from proliferation.

### 3.4. Cytomorphological and Inflammatory Responses After Scratching

**Figure 8:**
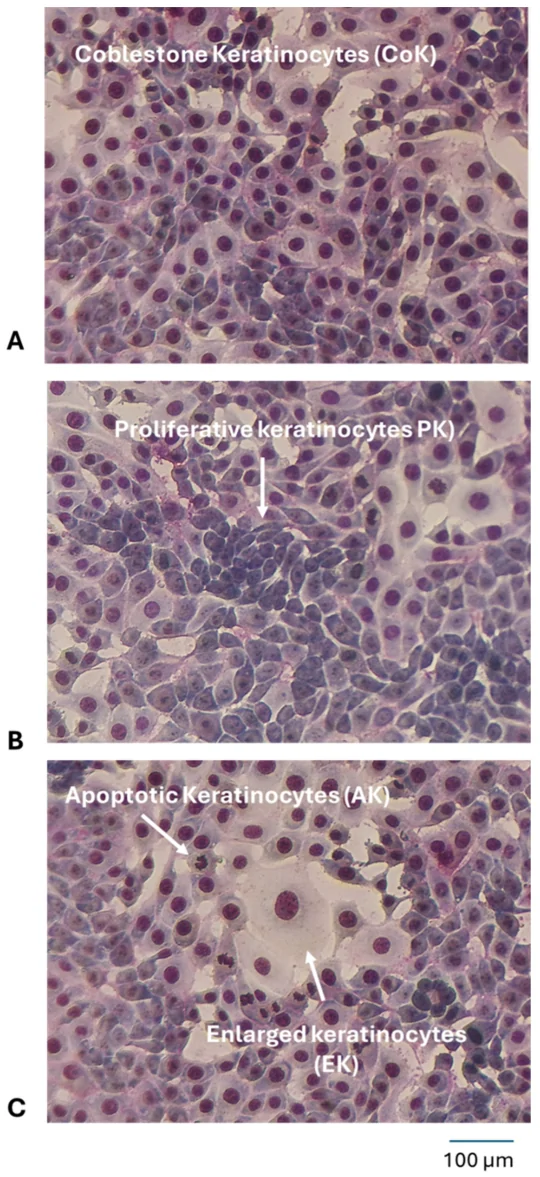
Representative morphology-based phenotypes in HaCaT cultures: (**A**) cobblestone keratinocytes; (**B**) proliferative-appearing keratinocytes; and (**C**) apoptotic-like morphology and enlarged keratinocytes. Scale bar = 100 µm. These operational categories were defined by cytomorphology and were not validated as molecular cell states.

**Figure 9:**
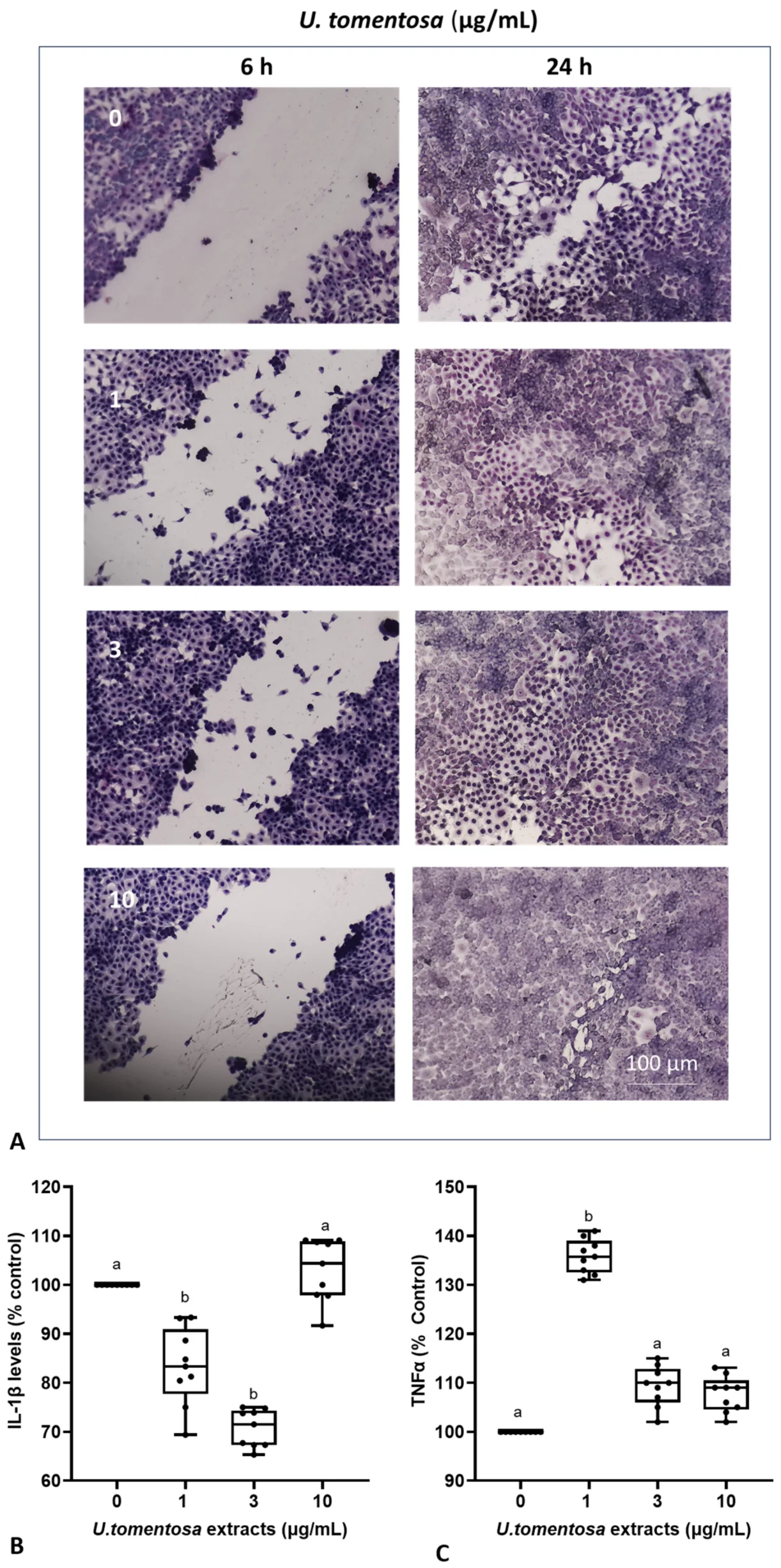
Morphology and inflammatory mediators following scratch injury (*scratch assay*). (**A**) Representative images of stained HaCaT cultures 6 and 24 h after treatment with *U. tomentosa* aqueous extract. (**B**) IL-1β and (**C**) TNF-α levels measured after 24 h of treatment. Each point represents the mean of technical replicates from an independent biological experiment (*n* = 3). Horizontal lines and error bars indicate the mean ± SD. Cytokine levels were normalized to the percentage of the untreated control. Data are presented as mean ± SD. Different letters indicate statistically significant differences between groups (*p* < 0.05), determined by one-way ANOVA followed by Tukey’s multiple-comparison test. Scale bar = 100 µm.

Morphology-based classification identified cobblestone, proliferative-appearing, apoptotic-like morphology, and enlarged keratinocyte phenotypes (Figure 8). At 24 h after scratching, cobblestone keratinocytes were the predominant phenotype across groups. The distributions of cobblestone, proliferative-appearing, and apoptotic-like morphology cells did not differ significantly (Table 3; Figure 9). Enlarged keratinocytes were more frequent at 1 than at 10 µg/mL; the control and 3 µg/mL groups were intermediate and did not differ from either group. Cell enlargement alone is not sufficient to identify senescence and may also reflect stress, spreading, differentiation, or wound-edge leader-cell behavior.

IL-1β concentrations decreased at 1 and 3 µg/mL relative to the control, with the lowest value at 3 µg/mL; the 10 µg/mL group returned toward the control value. TNF-α showed only modest, nonmonotonic variation without a consistent difference from control (Figure 8). Thus, the extract selectively altered IL-1β rather than broadly suppressing inflammatory signaling.

## 4. Discussion

The principal finding of this study was that an aqueous *U. tomentosa* stem-bark extract enhanced scratch closure in HaCaT keratinocyte monolayers without compromising metabolic viability or membrane integrity under the conditions tested. Two complementary aspects warrant discussion: the chemical profile of the aqueous preparation and the cellular responses accompanying enhanced closure, including selective changes in inflammatory mediators, redox-related endpoints, and cell-cycle distribution.

Chemical characterization of the aqueous *U. tomentosa* stem-bark extract revealed a diverse profile, with putative annotations encompassing flavonoid glycosides, proanthocyanidins, caffeoylquinic-acid derivatives, iridoid/secoiridoid glycosides, and triterpenoid glycosides. These findings complemented the colorimetric detection of phenolic compounds, flavonoids, condensed tannins, and alkaloids. Among the annotated flavonoids, rutin is particularly relevant to the discussion of wound repair. The existing literature provides biological plausibility for investigating whether the constituents of the aqueous extract contribute to the accelerated closure of scratch assays (cell migration assays) in keratinocytes [35].

Proanthocyanidins represent another chemical class relevant to the interpretation of the extract profile, which included putatively annotated procyanidin B1 and colorimetrically detected condensed tannins. Prokop et al. [36] reviewed preclinical evidence linking proanthocyanidins to wound repair through antioxidant, anti-inflammatory, pro-regenerative, and pro-angiogenic activities. These findings provide a rationale for investigating their potential contribution to the responses observed here. However, evidence obtained with proanthocyanidin-rich preparations cannot be directly attributed to procyanidin B1 or to the present extract, and the involvement of these constituents in enhanced scratch closure remains untested.

The putative annotation of iridoid and secoiridoid glycosides also merits consideration. Öztürk et al. (2006) [37] reported that sweroside stimulated collagen production in cultured chicken embryonic fibroblasts, supporting its potential relevance to tissue repair. More recently, the authors of [38] developed a pH-responsive chitosan/carboxymethyl cellulose hydrogel containing geniposidic acid and reported accelerated wound healing in an animal model. Together, these studies support further investigation of selected iridoid and secoiridoid glycosides in reparative applications. However, differences in cell models, delivery systems, and experimental endpoints preclude direct extrapolation to the enhanced HaCaT scratch closure observed here, and the contribution of these constituents to the activity of our extract remains untested.

The putative annotation of triterpenoid glycosides also warrants consideration, as selected members of this class have demonstrated wound-repair activity. Bandopadhyay et al. (2023) [39] reviewed the wound-healing, anti-inflammatory, and antioxidant properties of asiaticoside and madecassoside, two triterpenoid glycosides from *Centella asiatica*. More recently, Sucharitakul et al. (2026) [40] demonstrated that madecassoside enhanced scratch closure in HaCaT monolayers, with pharmacological evidence implicating Ca^2+^/AMPK- and mTOR-dependent ERK signaling. Complementary evidence is provided by Yang et al. (2023) [41], who reviewed polymer-based wound dressings incorporating ginsenoside Rg3 and discussed their potential to promote wound repair through mechanisms including inflammatory and oxidative-stress modulation. Together, these reports provide a biological rationale for investigating the putatively annotated triterpenoid glycosides in our aqueous *U. tomentosa* extract. However, their individual identities and contributions to the observed closure response remain to be established.

Although the extract enhanced closure under direct exposure in keratinocyte monolayers, its local availability following topical application remains to be established. Disruption of the epidermal barrier in an open wound may facilitate access to exposed tissue but does not ensure effective delivery of all extract constituents. Future studies should therefore assess formulation-dependent release, stability, and local retention, together with repair-related activity in more complex skin models.

The principal finding of this study was that the aqueous *U. tomentosa* stem-bark extract enhanced scratch closure in HaCaT keratinocyte monolayers while preserving metabolic viability and membrane integrity across the tested concentration range. The chemical profile and the accompanying inflammatory, redox, and cell-cycle responses provide complementary perspectives on this functional effect. However, these associations do not establish which constituents or cellular pathways were responsible for enhanced closure.

The first aspect to consider is the chemical composition of the aqueous extract. The aqueous extract contained putatively annotated flavonoid glycosides, proanthocyanidins, caffeoylquinic-acid derivatives, iridoid/secoiridoid glycosides, and triterpenoid-related features in addition to alkaloid-related classes. Several of these compound families have been associated with antioxidant or inflammation-modulating activities [10,12,13]. As discussed above, experimental evidence for selected representatives also supports their potential involvement in repair-related processes. Nevertheless, studies of isolated compounds or formulated products cannot be directly extrapolated to this chemically complex extract. Extraction conditions may influence the recovered chemical profile, making preparation-specific characterization particularly relevant when comparing aqueous and hydroalcoholic extracts. The results should not, however, be attributed to any individual metabolite because the annotations were not confirmed with authentic standards and no bioactivity-guided fractionation was performed. Likewise, additive or synergistic interactions among constituents remain hypotheses requiring experimental evaluation.

The second aspect concerns the enhanced closure response observed at all tested scratch-assay concentrations at 6 h and at 3 and 10 µg/mL at 24 h. The response at 6 h suggests an early effect on closure dynamics, but its timing does not identify the relative contributions of movement, spreading, and proliferation. However, an important limitation of the present study is the relatively small number of independent biological replicates (*n* = 3). Therefore, the scratch assay has limitations that should be considered when interpreting these findings. Separate cultures were assessed at each time point, and closure percentages were estimated using the initial cell-free area of independent untreated control cultures as a common reference. Consequently, differences in initial scratch dimensions among treatment groups could not be accounted for and may have contributed to the observed differences. Although the Shapiro–Wilk test did not detect a significant departure from normality in the model residuals, the small number of independent experiments (*n* = 3 per group) limits the assessment of statistical assumptions and the precision of effect estimates. These results therefore warrant confirmation in additional independent experiments with individual baseline measurements for each well.

The scratch assay integrates collective cell movement, spreading, and proliferation [33,34]. Because proliferation was not experimentally blocked, the present data should be interpreted as enhanced monolayer closure rather than migration alone. Cell-cycle data provide useful context but do not resolve these processes completely. Future live-cell tracking or scratch assays performed under validated proliferation-limiting conditions would distinguish these contributions.

In contrast to the enhanced scratch closure observed in the present study at relatively low concentrations (1–10 µg/mL), Detering et al. (2025) [15] reported impaired wound closure in HaCaT keratinocytes exposed to hydroethanolic *U. tomentosa* extracts. Their experimental protocol evaluated botanical extracts over a concentration range of 5–80 µg/mL; however, the comparative wound-healing results were reported for a selected “optimal concentration,” and the specific concentration of *U. tomentosa* corresponding to the reported response was not specified. Thus, a direct concentration-matched comparison with the present findings cannot be established. Notably, the 1 and 3 µg/mL concentrations evaluated here were below the lowest concentration included in their reported experimental range. In addition to these concentration-related differences, extract preparation may also be relevant, as the present study employed an aqueous extract whereas Detering et al. evaluated extracts prepared with 20% and 70% ethanol. These preparations are expected to differ in their chemical profiles; however, whether differences in extraction solvent and resulting composition account for the contrasting effects on scratch closure remains to be established through direct comparison of chemically characterized preparations.

Enhanced closure was accompanied by selective modulation of inflammatory readouts. Inflammatory signaling must be appropriately timed during repair. IL-1β and TNF-α activate keratinocytes and contribute to early wound responses, whereas prolonged cytokine production can maintain tissue injury and delay closure [2,3,4]. The reduction in IL-1β at 1 and 3 µg/mL, together with broadly stable TNF-α, indicates a selective response within the measured cytokine panel. These findings are insufficient to establish generalized suppression of inflammatory signaling. Furthermore, the concentration–response patterns for IL-1β and closure were not fully aligned: enhanced closure persisted at 10 µg/mL, whereas a reduction in IL-1β was observed at the lower concentrations. Thus, reduced IL-1β may accompany the repair-related response but cannot, on its own, explain it.

The limited changes in basal redox readouts and the preservation of viability and membrane integrity also inform interpretation of the closure response. Under the conditions tested, enhanced closure occurred without evidence of marked basal redox imbalance or overt cytotoxicity. However, the absence of substantial changes in these readouts does not demonstrate antioxidant protection under an oxidative challenge, nor does it exclude changes in localized or transient redox signaling. Similarly, the nonmonotonic redistribution of cell-cycle phases should not be interpreted as direct evidence of increased proliferation. Measurements of DNA synthesis, cell number, and time-resolved cell-cycle progression would help establish its functional significance.

Extracellular NDUFS7 should be interpreted separately as an exploratory readout. The absence of an increase does not establish preserved mitochondrial respiration or exclude mitochondrial stress, and the present findings do not demonstrate a mitochondrial mechanism for enhanced closure. Direct measurements of oxygen consumption, ATP production, mitochondrial membrane potential, and mitochondrial ROS would be needed to investigate this possibility. Overall, the data support enhanced closure accompanied by selective cellular responses, while leaving the causal relationships among these endpoints unresolved.

The findings are relevant to aging because inefficient re-epithelialization, chronic low-grade inflammation, redox dysregulation, and mitochondrial dysfunction are interconnected features of aged skin and impaired wound repair [1,2,3,4,5,6,7]. The IL-1β and TNF-α can occur within inflammatory networks that overlap with the SASP, while altered ROS handling, mitochondrial function, cell-cycle regulation, and enlarged morphology can accompany cellular senescence [5,6,7]. Nonetheless, none of these features is specific to senescence. The cultures were not subjected to a validated senescence-induction protocol, stable growth arrest was not demonstrated, and canonical markers such as SA-β-gal, p16INK4a, p21CIP1, persistent DNA-damage foci, or a broader secretome panel were not measured. Accordingly, this study does not demonstrate a senomorphic, senolytic, or SASP-modulating effect. Instead, it identifies repair-related and inflammatory responses that warrant investigation in models specifically designed to examine skin aging and inflammaging.

The aqueous preparation provides a connection with traditional botanical use, but the route of exposure remains an important translational distinction. These constituents should not be described collectively as essential nutrients, and the current in vitro exposure does not establish an effect of tea consumption. Gastrointestinal transformation, absorption, host and microbial metabolism, circulating conjugates, and delivery to skin may substantially alter exposure. Simulated digestion, intestinal transport, pharmacokinetic studies, and oral in vivo models will be necessary before nutritional or systemic claims can be made.

Topical application would require a separate assessment of local delivery. Although disruption of the epidermal barrier in an open wound may facilitate access to exposed tissue, it does not ensure effective delivery of all extract constituents. Formulation-dependent release, stability, and local retention therefore remain relevant. Because the present model directly exposed keratinocytes to the extract, it does not establish cutaneous penetration, tissue availability, or the efficacy of a topical preparation [42].

Several limitations require emphasis. HaCaT cells are immortalized and do not fully model primary keratinocytes, aged skin, immune-cell interactions, vascular contributions, or the three-dimensional wound environment. The scratch assay assessed monolayer closure without separating migration from proliferation, and the measured cytokines provide only a limited view of inflammatory signaling. The cytomorphological categories were operational and require validation. The exploratory nature of extracellular NDUFS7 and the putative metabolomic annotations further limit mechanistic attribution. Future work should combine primary keratinocytes from donors of different ages, keratinocyte-fibroblast or three-dimensional skin models, validated senescence induction, multiplex SASP profiling, mitochondrial respiration, and bioavailability studies. Bioactivity-guided fractionation and confirmation of selected chemical annotations would additionally help connect the extract composition to its functional effects.

## 5. Conclusions

A chemically diverse aqueous *U. tomentosa* stem-bark extract enhanced the closure of scratched HaCaT keratinocyte monolayers and selectively modulated IL-1β, cytomorphology, cell-cycle distribution, and redox-metabolic endpoints without overt cytotoxicity. These findings support the potential of this aqueous preparation to influence cellular responses relevant to epidermal repair. However, the associations among the measured endpoints do not establish the mechanisms underlying enhanced closure or distinguish the contributions of migration and proliferation.

The putatively annotated chemical constituents provide candidates for further investigation, although the observed activity cannot be attributed to individual metabolites or interactions among them. Confirmation of metabolite identities, bioactivity-guided fractionation, and evaluation in primary keratinocytes and three-dimensional skin models would help clarify the relationship between extract composition and repair-related activity.

The results support further investigation of *U. tomentosa*-derived bioactives in epidermal repair and age-relevant skin models. Their relevance to inflammaging remains a rationale for future research, as the present study does not demonstrate modulation of cellular senescence or the senescence-associated secretory phenotype (SASP). Likewise, direct exposure of keratinocytes to the extract does not establish efficacy after oral consumption or topical application. Studies in validated aging and senescence models, together with route-specific assessments of bioavailability and delivery, are needed to advance its potential application.

## Figures and Tables

**Table 1:** Colorimetric characterization and DPPH-scavenging activity of the aqueous *U. tomentosa* extract.

Analysis	Result
Total phenolic content	236.87 ± 0.02 mg GAE/g
Flavonoids	19.59 ± 0.01 mg QE/g
Condensed tannins	12.16 ± 0.04 mg CE/g
Alkaloids	5.84 ± 0. 6 mg BNE/g
DPPH	IC50 = 148.5 ± 0.09 µg/mL

Values are mean ± SD from three replicate measurements. GAE: gallic acid equivalents (Folin–Ciocalteu method); QE: quercetin equivalents (aluminum chloride method); CE: catechin equivalents (vanillin–HCl method); BNE/g: bismuth nitrate equivalents per gram (spectrophotometric Dragendorff method). Values are expressed per gram of dried extract (mean ± standard deviation). IC50, concentration producing 50% inhibition.

**Table 2:** Representative metabolites putatively annotated by LC-HRMS/MS and spectral matching *.

Putative Annotation	Measured *m*/*z*	Molecular Formula	Adduct	Cosine
Sweroside	403.1243	C_16_H_22_O_9_	[M+FA-H]^−^	0.77
Loganic acid	375.1291	C_16_H_24_O_10_	[M-H]^−^	0.90
Taxifolin-3-glucoside	465.1033	C_21_H_22_O_12_	[M-H]^−^	0.88
Rutin	609.1460	C_27_H_30_O_16_	[M-H]^−^	0.98
Quercetin-3-O-glucoside	463.0878	C_21_H_20_O_12_	[M-H]^−^	0.98
Procyanidin B-type	577.1346	C_30_H_26_O_12_	[M-H]^−^	0.90
Neochlorogenic acid	353.0872	C_16_H_18_O_9_	[M-H]^−^	0.96
Afzelin	431.0980	C_21_H_20_O_10_	[M-H]^−^	0.95
Kaempferol-3-O-glucoside	447.0928	C_21_H_20_O_11_	[M-H]^−^	0.80
Isochlorogenic acid C	515.1189	C_25_H_24_O_12_	[M-H]^−^	0.97
Geniposidic acid	419.1190	C_16_H_22_O_10_	[M+FA]^−^	0.80
Chlorogenic acid	353.0873	C_16_H_18_O_9_	[M-H]^−^	0.96

* The metabolites shown here represent a selected subset of the chemical profile detected by LC-MS/MS and were chosen to highlight the main chemical classes and the most informative annotations. The complete set of putatively annotated metabolites is provided in Table S1 of the Supplementary Material. Additional analytical parameters used to support metabolite annotation, including retention time, theoretical and measured *m*/*z* values, mass error, MS/MS product ions, molecular formula, adduct assignment, and spectral similarity scores, are also reported in Table S1.

**Table 3:** Morphology-based phenotype distribution 24 h after scratching.

Extract (µg/mL)	Cobblestone (%)	Proliferative-Appearing (%)	Apoptotic-Lik Morphology (%)	Enlarged (%)
0	39.0 ± 13.7 ^a^	11.4 ± 4.0 ^a^	37.4 ± 20.1 ^a^	12.1 ± 25.2 ^ab^
1	46.3 ± 5.0 ^a^	6.7 ± 2.5 ^a^	15.4 ± 2.0 ^a^	31.5 ± 9.6 ^a^
3	63.6 ± 10.1 ^a^	10.1 ± 2.9 ^a^	17.7 ± 4.7 ^a^	8.6 ± 4.9 ^ab^
10	61.1 ± 6.4 ^a^	12.0 ± 3.1 ^a^	20.0 ± 8.0 ^a^	6.8 ± 2.0 ^b^

Values are mean ± SD. Within a column, groups that do not share a letter differ at *p* < 0.05 by one-way ANOVA followed by Tukey’s test. The cytomorphological analysis was performed in triplicate for each treatment, with at least 10 microscopic fields evaluated per treatment at 20× magnification.

## Data Availability

The raw biological data, LC-MS/MS datasets, GNPS molecular-networking task, and associated analysis files supporting the findings of this study are available from the corresponding author upon reasonable request.

## Supplementary Materials

The following supporting information can be downloaded at: https://www.mdpi.com/article/10.3390/nu18213715/s1, Figure S1: Feature-Based Molecular Networking (FBMN) of metabolites analyzed by LC–MS/MS and processed through the GNPS platform. Each node represents a molecular feature, and connected nodes are grouped according to MS/MS spectral similarity. Polygon-shaped nodes indicate features with spectral matches to the GNPS library, whereas circular nodes represent unannotated features. Node colors indicate the assigned Natural Products (NP) pathway: green, shikimates and phenylpropanoids; yellow, fatty acids; purple, terpenoids; teal, alkaloids; brown, carbohydrates; and light pink, unclassified features. Table S1: Annotation of metabolites based on LC-HRMS/MS data and spectral matching in the GNPS platform.
