# Supplementary material for: Chemical Characterization and Effects of Aqueous Uncaria tomentosa Stem-Bark Extract on Keratinocyte Scratch Closure and Inflammatory and Redox Responses
Source: Nutrients. 2026 Nov 9;18(21):3715. doi: 10.3390/nu18213715 (PMC13766030; doi:10.3390/nu18213715)
Supplement: Supplementary file 1 [file nutrients-18-03715-s001.zip › nutrients-4593967-supplementary.pdf]

SUPPLEMENTARY MATERIAL

1. Dereplication and metabolite annotation workflow

Because no single annotation strategy can adequately cover the chemical diversity of a complex botanical extract, metabolite annotation was performed using a complementary, multi-evidence workflow. The different computational tools were therefore used to address distinct and complementary levels of chemical information, ranging from spectral relationships and molecular-formula assignment to structure candidates and chemical-class prediction.

1. MZmine processing: Chromatographic features were detected, deconvoluted, aligned, and grouped according to isotope and adduct relationships. This step generated the feature table and the corresponding MS/MS spectra used in the subsequent annotation workflows.

2. *GNPS: spectral evidence and molecular relationships:* The processed features were submitted to Feature-Based Molecular Networking (FBMN), in which nodes were organized according to MS/MS spectral similarity. GNPS spectral-library searching provided experimental reference matches for features represented in available libraries, whereas molecular networking enabled unannotated or partially annotated features to be grouped with structurally related metabolites based on shared fragmentation behavior (Figure S1).

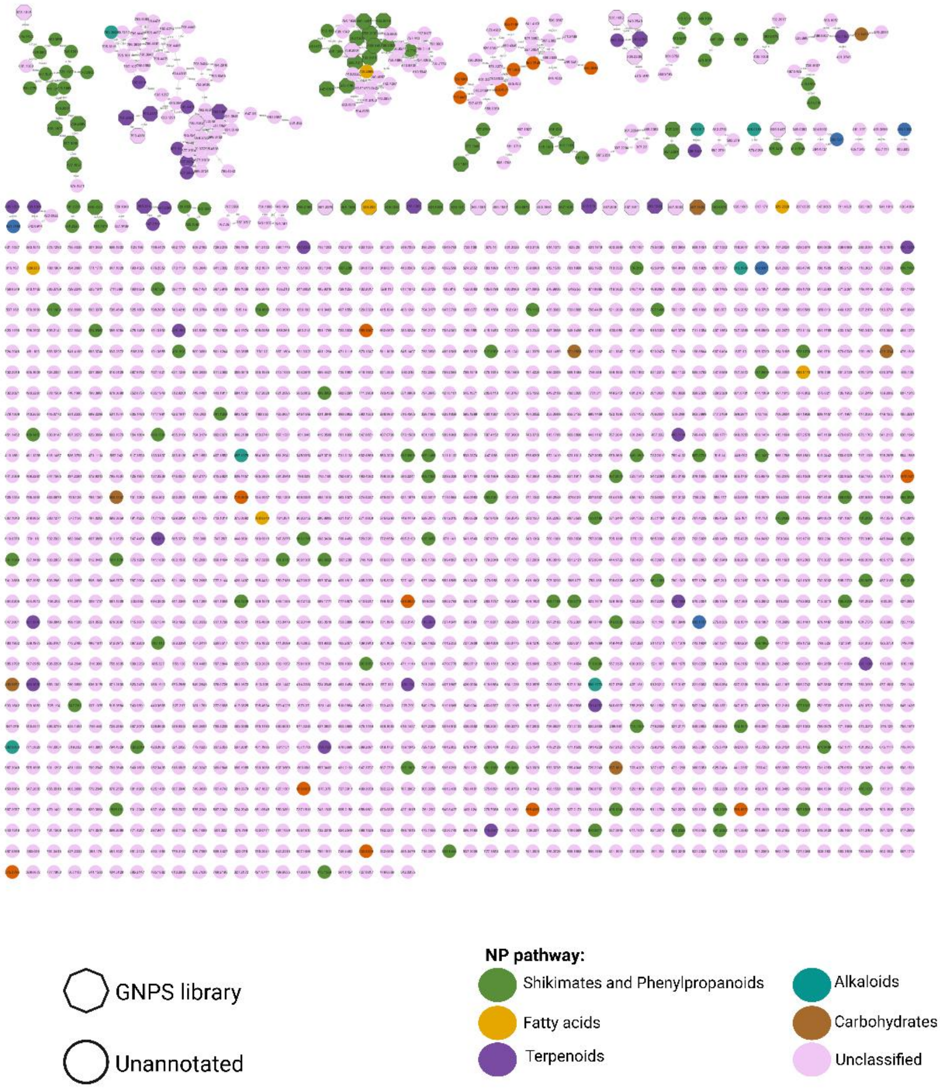

Figure S1: Feature-Based Molecular Networking (FBMN) of metabolites analyzed by LC–MS/MS and processed through the GNPS platform. Each node represents a molecular feature, and connected nodes are grouped according to MS/MS spectral similarity. Polygon-shaped nodes indicate features with spectral matches to the GNPS library, whereas circular nodes represent unannotated features. Node colors indicate the assigned Natural Products (NP) pathway: green, shikimates and phenylpropanoids; yellow, fatty acids; purple, terpenoids; teal, alkaloids; brown, carbohydrates; and light pink, unclassified features.

3. SIRIUS: molecular-formula assignment: Although GNPS spectral-library matching provided experimental support for several metabolite annotations, a substantial fraction of the detected MS/MS features could not be resolved through direct library comparison

alone. This limitation is inherent to untargeted natural-products metabolomics, since available spectral libraries represent only a fraction of the chemical diversity present in complex botanical matrices. To extend annotation coverage beyond compounds represented in experimental libraries, the MS/MS data were therefore further processed using the SIRIUS workflow. SIRIUS provided molecular-formula predictions from accurate-mass and fragmentation information and served as the computational framework for subsequent ZODIAC formula refinement, CSI structural annotation, FragHub spectral-library searching, and CANOPUS chemical-class prediction. Thus, the SIRIUS-based workflow complemented the GNPS results by enabling additional levels of chemical interpretation for features lacking direct or high-confidence spectral-library matches.

4. ZODIAC: molecular-formula refinement: The molecular-formula candidates generated by SIRIUS were further re-ranked using ZODIAC, which considers relationships among molecular formulas across the dataset to improve confidence in formula assignment.

4. CSI:FingerID: in silico structural annotation: For features with suitable molecular-formula assignments, CSI was used to predict molecular fingerprints from the experimental MS/MS spectra and rank candidate molecular structures available in structural databases. These results were interpreted as structural hypotheses rather than definitive compound identifications.

5. FragHub: complementary experimental spectral evidence: To expand the spectral-reference space beyond the libraries directly accessed through GNPS, the negative-ion FragHub resource integrated into SIRIUS was used for additional spectral-library searching. This step provided complementary experimental MS/MS evidence from harmonized mass spectral libraries.

6. CANOPUS: chemical-class assignment: For features for which compound-level structural annotation remained uncertain, CANOPUS was used to predict chemical classes from MS/MS-derived molecular information. CANOPUS assignments were also used to characterize the broader chemical landscape of the extract at the pathway, superclass, and class levels. Considering only assignments with an NP Class probability ≥ 0.75, a total of 560 chemical-class assignments were retained across the different levels of the NPClassifier hierarchy. Importantly, this number does not represent 560 individual metabolites, but rather hierarchical classifications associated with the detected molecular features, since a single feature may contribute to multiple levels of the classification system. Thus, this step provided a broader view of the chemical space represented in the

extract, extending the interpretation beyond the subset of features for which compound-level annotations could be proposed. These class-level assignments should therefore be interpreted as complementary chemical information rather than as individual metabolite identifications.

7. Integration and manual curation of annotation evidence: Final metabolite annotations were based on the convergence of accurate mass, molecular-formula assignment, ZODIAC re-ranking, MS/MS fragmentation, GNPS and FragHub spectral-library matches, CSI structural candidates, CANOPUS chemical-class predictions, and comparison with databases and previously reported metabolites from Uncaria species. Thus, annotation confidence was established from a body-of-evidence approach rather than from any single computational score or platform.

1.1 ***Feature-based molecular networking analysis and metabolite annotation***

To evaluate the chemical composition of the crude extract of *U. tomentosa,* the files quantification table of the features (.csv) and MS/MS file (.mgf) processed from Mzimine 4.10.6 were exported and processed to create a Feature-Based Molecular Networking (FBMN) workflow on the GNPS platform.

Molecular network was generated using a precursor ion mass tolerance of 0.02 Da and a fragment ion tolerance of 0.02 Da. Edges were retained when spectra shared a cosine score ≥ 0.75 with at least 4 matched fragment ions, whereas spectral library matches required a cosine score of 0.75 with a minimum of 3 matched peaks. The maximum molecular family size was set to 100 nodes, and the top 10 most similar neighbors for each node were retained. No intensity normalization was applied prior to network generation. The molecular networking is disponible in https://gnps2.org/status?task=cd90fd0c11a046efad2132315e0f9e1a. After, the molecular networking was exported to visualization using the Cytoscape software (version 3.10.4, Cytoscape Consortium, San Diego, CA, United States).

The annotation of metabolites was conducted according to the annotation levels reported by Metabolomics Standard Initiative (MSI) [Sumner et al., 2007]. The metabolites with library matching in GNPS were cured manually, comparing the mirror matching of the library compound and those obtained from extract, and performing a search for these metabolites in *Uncaria* species already reported in the literature and in database such as Pubchem, Mass Bank, Mona.

The MS/MS data were processed using SIRIUS software (version 6.4.0), applying an integrated workflow for molecular formula prediction, structure annotation, and

chemical class assignment. Molecular formula annotation was performed using the SIRIUS molecular formula identification workflow with the “de novo + bottom-up” strategy. The analysis was configured for a QTOF mass analyzer with an MS/MS mass accuracy of 10 ppm. The elemental composition was restricted to H, C, N, O, P, and S. For molecular formula generation, the allowed fallback adducts included $[M-H]^-$, $[M-H + FA]^-$, $[M+Cl]^-$, and $[M+Br]^-$. De novo formula generation was applied to features below m/z 400. Molecular formula candidates were subsequently re-ranked using ZODIAC based on molecular formula relationships within the feature network. Molecular structure annotation and compound class prediction were subsequently performed using CSI:FingerID. Candidate structures were ranked according to the molecular fingerprints predicted from the experimental MS/MS fragmentation patterns. The CSI search was configured with PubChem as a fallback database, using the approximate confidence mode and applying the score threshold implemented in SIRIUS. Analog searching was also enabled to expand the structural annotation beyond exact database matches. Chemical class prediction was performed using CANOPUS based on the molecular fingerprints generated during the SIRIUS workflow. This step was used to assign putative chemical classes to features for which a specific molecular structure could not be confidently assigned.

To complement the *in silico* annotation workflow, the spectral library search in SIRIUS was performed using the integrated fraghuneg2.siriusdb database, corresponding to the FragHub spectral resource configured for negative-ion MS/MS data. BioCyc was also enabled as a database resource within the SIRIUS workflow. Spectral-library searches were performed using a precursor mass deviation of 10 ppm. The integration of the FragHub resource allowed experimental MS/MS spectra to be compared against a harmonized collection of spectral records in addition to the structure- and formula-based annotation provided by SIRIUS.

The FragHub workflow is designed to integrate and harmonize mass spectral libraries from different sources, standardizing chemical identifiers and spectral metadata and allowing spectra to be separated according to chromatographic technique and ionization mode. In the present study, the negative-ion FragHub resource was therefore used to complement the SIRIUS-based annotation of the *U. tomentosa* extract. The use of integrated spectral libraries provides additional experimental spectral-matching evidence to the molecular formula, predicted structure, and chemical-class information generated by SIRIUS.

The final metabolite annotation was based on the combined evidence obtained from accurate mass measurements, molecular formula prediction, ZODIAC re-ranking, CSI structural prediction, spectral library matching, and CANOPUS chemical-class prediction. As the compounds were not confirmed using authentic analytical standards, they were considered putatively annotated rather than unequivocally identified.

2. CHEMICAL PROFILE

2.1 LC–MS/MS chemical profile of *U. tomentosa*

A total of 44 molecular features were selected for detailed assessment using the combined dereplication workflow, as shown in Table S1.

A substantial proportion of the annotated features corresponded to flavonoids and their glycosylated derivatives. Among the well-supported annotations were rutin, quercetin-3-*O*-glucoside, kaempferol-3-*O*-glucoside, afzelin, taxifolin-3-glucoside, and several di- and triglycosylated derivatives of quercetin and kaempferol. For example, the feature detected at *m/z* 609.1460 was annotated as rutin, with a GNPS cosine similarity of 0.986, and produced a prominent fragment at *m/z* 301.0350, consistent with the quercetin aglycone after cleavage of the disaccharide moiety. Likewise, quercetin-3-*O*-glucoside was detected at *m/z* 463.0878 and generated the characteristic aglycone ion at *m/z* 301.0342. The occurrence of multiple ions yielding fragments around *m/z* 301 further supported the presence of a chemically diverse series of quercetin glycosides rather than isolated individual compounds.

Kaempferol-derived metabolites were similarly well represented. Kaempferol-3-*O*-glucoside, afzelin, kaempferol rutinosides, and more highly glycosylated derivatives were detected in the extract. Their MS/MS spectra commonly generated product ions around *m/z* 285.04, corresponding to the deprotonated kaempferol aglycone, along with neutral losses consistent with hexose and deoxyhexose moieties. The presence of several features sharing the same aglycone but differing in precursor mass and chromatographic retention supports the occurrence of structurally diverse kaempferol glycosides within the flavonoid fraction of the extract.

The dereplication workflow also revealed proanthocyanidin-related features. A procyanidin dimer and procyanidin B1 were annotated at precursor masses around *m/z* 577.13. The MS/MS spectrum assigned to procyanidin B1 displayed a complex fragmentation pattern including ions at *m/z* 425, 407, 289, 245, 205, and 125, consistent with the fragmentation behavior expected for condensed flavan-3-ol dimers. These findings indicate that, in addition to monomeric and glycosylated flavonoids, oligomeric

flavonoid- derived phenolic compounds also contribute to the chemical profile of the hydroalcoholic extract.

Another major group comprised hydroxycinnamic acid and quinic acid derivatives. Neochlorogenic acid, chlorogenic acid, cryptochlorogenic acid, isochlorogenic acid C, 1,3-dicaffeoylquinic acid, and a feruloylquinic acid isomer were among the putatively annotated metabolites. The three monocaffeoylquinic acid isomers, detected at similar precursor m/z values, showed closely related MS/MS spectra dominated by the diagnostic quinic acid fragment at *m/z* 191.055, together with caffeic acid-related product ions at *m/z* 179.034 and 135.045. Their different retention times support the presence of positional caffeoylquinic acid isomers in the extract. Similarly, isochlorogenic acid C and 1,3-dicaffeoylquinic acid exhibited precursor ions around *m/z* 515.119, supporting the occurrence of dicaffeoylquinic acid derivatives.

The occurrence of iridoid and secoiridoid glycosides was also supported by several spectral-library matches. Sweroside, loganic acid, geniposidic acid, and 6′-*O*-β-D-apiofuranosylsweroside were annotated, together with additional glycosylated monoterpene-related features. Loganic acid was detected at *m/z* 375.1291 with a cosine similarity of 0.902, whereas sweroside was observed as the formate adduct $[M+FA-H]^-$ at *m/z* 403.1243.

At higher *m/z* values, a group of features centered at approximately *m/z* 793.438 was putatively assigned to glycosylated pentacyclic triterpenoids. These features shared the molecular formula $C_{42}H_{66}O_{14}$ and generated highly similar MS/MS fragmentation patterns, including product ions near *m/z* 749, 587, 569, and 441. Their closely related precursor masses, fragmentation patterns, and distinct retention times suggest the occurrence of several isomeric or closely related triterpenoid glycosides. Library candidates were consistent with ursane/oleanane-type triterpenoid structures bearing deoxyhexose and hexose moieties. Because tandem mass spectra of pentacyclic triterpenoid isomers often provide limited information for distinguishing stereochemical configuration and glycosylation/substitution sites, these assignments were conservatively interpreted at the structural-family level rather than as specific compound-level annotations.

High spectral similarity was observed for several GNPS library matches. Several annotations exhibited cosine scores above 0.90, including rutin, quercetin-3-O-glucoside, chlorogenic acid, isochlorogenic acid C, afzelin, and several other glycosylated

metabolites. Accurate-mass agreement was also high, with mass errors ranging from −3.66 to +0.91 ppm, and the vast majority of values falling within approximately ±2 ppm.

Several features were additionally putatively assigned to lignan- or phenylpropanoid-derived glycosides. In particular, benzofuran-containing glycosides and methoxylated aromatic glycosides were represented among the spectral-library matches. Although some of these annotations were associated with generic database identifiers rather than well-established trivial names, their precursor masses, molecular compositions, and fragmentation patterns support the occurrence of oxygen-rich metabolites related to phenylpropanoid metabolism.

2.2 Chemical-class distribution and phytochemical interpretation

At the pathway level, shikimates and phenylpropanoids represented the largest fraction of the assignments (n = 254; 45.4%), followed by terpenoids (n = 115; 20.5%). Together, these two pathways accounted for approximately two-thirds of the retained classifications, indicating that phenolic and terpenoid-related metabolites constitute major components of the chemical space detected in the extract. The remaining assignments were distributed among alkaloids (n = 43; 7.7%), polyketides (n = 41; 7.3%), fatty acids (n = 40; 7.1%), amino acids and peptides (n = 35; 6.3%), and carbohydrates (n = 32; 5.7%).

Within the shikimates and phenylpropanoids pathway (Figure 2), flavonoids were particularly prominent (n = 145), followed by phenylpropanoids (C6–C3; n = 32), lignans (n = 19), phenylethanoids (C6–C2; n = 18), phenolic acids (C6–C1; n = 9), and stilbenoids (n = 6). At a more specific classification level, flavonols constituted the most abundant flavonoid group (n = 71), whereas other predicted classes included proanthocyanins (n = 16), flavones (n = 15), dihydroflavonols (n = 13), flavanones (n = 8), and anthocyanidins (n = 7).

Terpenoid-related metabolites formed the second major branch of the classification profile (Figure 3). Among these assignments, triterpenoids predominated (n = 66), followed by monoterpenoids (n = 21), diterpenoids (n = 13), and apocarotenoids (n = 6). The triterpenoid fraction was strongly represented by oleanane-type triterpenoids (n = 47), whereas the monoterpenoid branch included a marked contribution from iridoids (n = 15) and secoiridoids (n = 5).

Regarding the less represented contributors to the chemical profile of *U. tomentosa* (Figure 4), cinnamic acids and derivatives (n = 31) within the phenylpropanoid branch further reinforce the contribution of shikimate-derived metabolism to the chemical

complexity revealed by LC–MS/MS. Although alkaloids accounted for a smaller proportion of the overall CANOPUS assignments (7.7%) than shikimate/phenylpropanoid- and terpenoid-derived compounds (Figure 4), their detection remains particularly relevant in *U. tomentosa*, a species widely recognized for its structurally diverse alkaloid chemistry (Batiha et al., 2020). The comparatively lower numerical contribution of alkaloid-related classifications should therefore not be interpreted as indicating limited phytochemical importance. Rather, it reflects the broader chemical diversity captured by untargeted LC–MS/MS and by the hierarchical classification approach, in which numerous non-alkaloid constituents are simultaneously represented. Finally, the additional occurrence of polyketide-, fatty acid-, amino acid/peptide-, and carbohydrate-related assignments further demonstrates that the detectable metabolome comprises both specialized and more broadly distributed plant metabolites (Figure 4).

These results indicate that the chemical profile of the hydroalcoholic extract of *U. tomentosa* extends well beyond the alkaloid fraction traditionally emphasized for this species. This compositional complexity should also be considered when evaluating the bioactivity of *U. tomentosa*. Several studies have highlighted the contribution of non-alkaloid constituents to the pharmacological properties associated with *U. tomentosa*, reinforcing the importance of considering its broader phytochemical composition rather than focusing exclusively on alkaloids **(Navarro-Hoyos et al., 2017), (Dreifuss et al., 2013), (Arado et al., 2024), (Lopes et al., 2025)**.

Table 1: Annotation of metabolites based on LC-HRMS/MS data and spectral matching in the GNPS platform.

**Feature**	**Rt (min)**	**Theoretical mass *m/z***	**Measured *m/z***	**Error (ppm)**	**MS/MS**	**Molecular formula**	**Annotation**	**Adduct**	**Cosine**	**Ref**
**654**	29.37	403.12459	403.1243	-0.71	357.1189; 237.0763; 195.0655; 161.0431; 151.0751; 125.0239; 119.0352; 113.0241; 101.0239	$C_{16}H_{22}O_9$	Sweroside	[M+FA−H]⁻	0.777908	(Sun et al., 2024); (Fawaid & Mahfudh, 2025)
**35**	1.13	405.10144	405.1008	-1.59	192.0120; 191.05556; 173.0444; 127.0393; 111.0447	$C_7H_{12}O_6$	Quinic acid	[2M−2H+Na]⁻	0.950717	(Åkesson et al., 2005)
**431**	16.57	375.12967	375.1291	-1.52	213.0773; 169.0864; 151.0760; 113.0242	$C_{16}H_{24}O_{10}$	Loganic acid	[M−H]⁻	0.902361	Ferreira et al., 2026); (Wang et al., 2025)
**1316**	53.03	577.15628	577.1558	-0.83	432.5427; 431.0970; 430.0903; 285.0399; 283.0241; 255.0274	$C_{27}H_{30}O_{14}$	Lespenefril (kaempferitrin)	[M−H]⁻	0.960608	(Li et al., 2023); (Barboza et al., 2015)

**1329**	53.16	593.15119	593.151	-0.33	327.0504; 299.0563; 287.7491; 286.2081; 285.5718; 285.0397; 284.0322; 257.0454; 256.0380; 255.0295; 241.0507; 239.0349; 229.0499; 227.0349; 185.0627; 178.9966; 163.0028; 151.0031	$C_{27}H_{30}O_{15}$	Kaempferol-3-O-rutinoside	[M−H]⁻	0.974915	(Farag et al., 2013); (Shiyan et al., 2025)
**734**	32.4	465.10385	465.1033	-1.18	285.0392; 151.0034; 107.0131	$C_{21}H_{22}O_{12}$	Taxifolin-3-glucoside	[M−H]⁻	0.887523	14187089
**1202**	47.45	609.14611	609.146	-0.18	343.0448; 313.0329; 303.0696; 302.2299; 301.6466; 301.0350; 300.0270; 283.0231; 273.0397; 272.0324; 271.0239; 255.0292; 254.0262; 178.9982; 151.0028	$C_{27}H_{30}O_{16}$	Rutin	[M−H]⁻	0.986043	(Farag et al., 2013); (X. Zhang et al., 2022)
**1241**	48.18	593.15119	593.1507	-0.83	447.0921; 446.0849; 301.0350; 299.0191; 271.0231; 255.0308	$C_{27}H_{30}O_{15}$	Quercetin-3,7-O-alpha-L-dirhamnopyranoside	[M-H]-	0.957017	(Kachlicki et al., 2008); (da Paixão et al., 2021)

**814**	35.74	609.14610	609.1457	-0.67	489.1019; 463.0789; 462.0751; 447.0935; 446.0846; 301.0349; 285.0389; 283.0252;	—	Cyanidin-3,5-di-O-glucoside chlorid	[M-H]-	0.77755	164999
**1235**	47.9	463.0882	463.0878	-0.86	301.0342; 300.0270; 271.0244; 255.0287; 254.0205; 243.0294; 178.9981; 151.0032	$C_{21}H_{20}O_{12}$	Quercetin-3-O-glucoside	[M−H]⁻	0.988921	(Kachlicki et al., 2008); (X. Zhang et al., 2022)
**914**	40.26	625.14102	625.141	-0.04	463.0864; 301.0348; 300.0312	$C_{27}H_{30}O_{17}$	Quercetin 3,4'-diglucoside	[M−H]⁻	0.936276	5320835
**1222**	47.5	577.13515	577.1347	-0.78	425.0838; 407.0737; 289.0701; 125.0249	$C_{30}H_{26}O_{12}$	Procyanidin dimer	[M−H]⁻	0.867954	(Cunha et al., 2020); (Blanck et al., 2022)
**543**	22.6	577.13515	577.1346	-0.95	559.1209; 463.0852; 451.1026; 441.1014; 425.0878; 407.0770; 395.0947; 381.0970; 339.0869; 299.0568; 289.0714; 287.0567;	$C_{30}H_{26}O_{12}$	Procyanidin B1	[M−H]⁻	0.901084	(Blanck et al., 2022)

					245.0821; 205.0485; 203.0684; 161.0237; 125.0236					
**312**	10.23	353.08781	353.0872	-1.72	191.0558; 179.0345; 135.0450	$C_{16}H_{18}O_9$	Neochlorogenic acid	$[M-H]^-$	0.965173	(Chen et al., 2011); (Xu et al., 2023)
**614**	28.65	563.23453	563.234	-0.94	517.2303; 311.0977; 293.0889; 251.0754; 221.0659; 191.0553; 149.0447; 113.0235; 101.0239	$C_{24}H_{38}O_{12}$	4-[2-[(2S,3R,4S,5S,6R)-6-[[(2S,3R,4R)-3,4-dihydroxy-4-(hydroxymethyl)oxolan-2-yl]oxymethyl]-3,4,5-trihydroxyoxan-2-	$[M+FA]^-$	0.839536	45360003

							yl]oxy-4-hydroxy-2,6,6-trimethylcyclohexylidene]but-3-en-2-one			
**597**	28.24	447.1508	447.1503	-1.12	401.1447; 269.1025	$C_{18}H_{26}O_{10}$	Benzyl 2-O-beta-D-xylopyranosyl-beta-D-glucopyranoside	$[M+FA]^-$	0.940077	10763616
**571**	26.06	447.1508	447.1503	-1.12	401.1452; 269.1022; 161.0461	$C_{18}H_{26}O_{10}$	Benzyl 2-O-beta-D-xylopyranosyl-beta-D-glucopyranoside	$[M+FA]^-$	0.951407	10763616

**1273**	49.86	509.22397	509.2235	-0.91	463.2177; 331.1760; 161.0432; 149.0445; 131.0350; 101.0235	$C_{21}H_{36}O_{11}$	2-[[3,4-dihydroxy-4-(hydroxymethyl)oxolan-2-yl]oxymethyl]-6-[(1,3,3-trimethyl-2-oxabicyclo[2.2.2]octan-5-yl)oxy]oxane-3,4,5-triol	$[M+FA]^-$	0.903542	57509351
**1223**	47.63	593.15119	593.1508	-0.66	285.0403; 284.0320; 255.0287; 227.0356	$C_{27}H_{30}O_{15}$	Kaempferol 7-	$[M-H]^-$	0.901397	5483905

							neohesperidoside			
**1426**	58.47	537.16137	537.1611	-0.49	373.1296; 331.1214; 179.0347; 161.0237; 135.0449	$C_{25}H_{30}O_{13}$	(2S,3S,4S)-4-[2-[(E)-3-(3,4-dihydroxyphenyl)prop-2-enoyl]oxyethyl]-3-ethenyl-2-[(2S,3R,4S,5S,6R)-3,4,5-trihydroxy-6-(hydroxymethyl)oxan-2-yl]oxy-	[M−H]⁻	0.953593	95223113

							3,4-dihydro-2H-pyran-5-carboxylic acid			
**1372**	55.01	549.25527	549.2549	-0.66	503.2490; 371.2075; 209.1547; 161.0444	$C_{24}H_{40}O_{11}$	3,5,5-trimethyl-4-[3-[(2R,3R,4S,5S,6R)-3,4,5-trihydroxy-6-[[(2S,3R,4S,5R)-3,4,5-trihydroxyoxan-2-yl]oxymeth	[M+FA−H]−	0.896881	45360253

							yl]oxan-2-yl]oxybutyl]cyclohex-2-en-1-one			
**702**	31.63	363.10854	363.1081	-1.21	315.0850; 256.0721; 167.0354; 165.0554; 150.0314	$C_{18}H_{20}O_8$	3-methoxy-4-[2-methoxy-4-(1,2,3-trihydroxypropyl)phenoxy]benzoic acid	$[M-H]^-$	0.815725	51136371
**1453**	58.71	477.10385	477.1037	-0.31	431.0970; 285.0396; 284.0329; 255.0292; 229.0476	$C_{21}H_{20}O_{10}$	Afzelin	$[M+FA-H]^-$	0.956186	5316673
**738**	32.59	461.16645	461.1658	-1.41	415.1604; 269.1019; 101.0238	$C_{19}H_{28}O_{10}$	Hydrangeifolin I	$[M+FA-H]^-$	0.918222	10549806
**756**	33.52	367.10346	367.1029	-1.51	191.0557; 134.0368	$C_{17}H_{20}O_9$	Feruloyl quinic acid	$[M-H]^-$	0.889576	(da Silva Pontes et al., 2024)

							(isomer of 886, 888)			
**1063**	43.14	609.14611	609.1455	-1	463.0868; 446.0849; 301.0356; 300.0268; 299.0191; 271.0220; 255.0308; 178.9984	$C_{27}H_{30}O_{16}$	Quercetin 3-(2-glucosylrhamnoside)	[M−H]⁻	0.94229	14238625
**1073**	43.56	755.20402	755.2037	-0.42	609.1457; 447.0928; 446.0854; 301.0355; 300.0266; 299.0191	$C_{33}H_{40}O_{20}$	Quercetin-3-O-alpha-L-rhamnopyranosyl(1-2)-beta-D-glucopyranoside-7-O-alpha-L-rhamnopyranoside	[M−H]⁻	0.865097	134776243
**1036**	42.18	627.22944	627.229	-0.71	581.2232; 419.1705; 405.1554; 404.1489; 233.0789	$C_{28}H_{38}O_{13}$	(2R,3R,4S,5S,6R)-2-[[6-	[M+FA]⁻	0.918313	24121292

							hydroxy-4-(4-hydroxy-3,5-dimethoxyphenyl)-3-(hydroxymethyl)-5,7-dimethoxy-1,2,3,4-tetrahydronaphthalen-2-yl]methoxy]-6-(hydroxymethyl)oxane-3,4,5-triol			
**941**	41.06	627.22944	627.2291	-0.55	582.8868; 582.7377; 581.2235; 419.1692; 405.1495; 404.1469;	$C_{28}H_{38}O_{13}$	(2R,3R,4S,5S,6R)-2-	[M+FA]⁻	0.872455	24121292

	401.1603; 386.1371; 371.1154; 233.0796; 115.0392	[[6-hydroxy-4-(4-hydroxy-3,5-dimethoxyphenyl)-3-(hydroxymethyl)-5,7-dimethoxy-1,2,3,4-tetrahydronaphthalen-2-yl]methoxy]-6-(hydroxymethyl)oxane-3,4,5-triol	

**878**	38.83	565.25018	565.2497	-0.85	520.8305; 520.8076; 520.5802; 519.2444; 387.2010; 293.0878; 233.0657; 225.1491; 191.0561; 161.0454; 153.0922; 125.0236	$C_{24}H_{40}O_{12}$	cis-(4S,5R)-4-hydroxy-3,3,5-trimethyl-4-[(E,3R)-3-[(2R,3R,4S,5S,6R)-3,4,5-trihydroxy-6-[[(2S,3R,4S,5R)-3,4,5-trihydroxyoxan-2-yl]oxymethyl]oxan-2-yl]oxybut-	[M+FA]⁻	0.88559	10839700

							1-enyl]cyclohexan-1-one			
**1345**	53.7	447.09329	447.0928	-0.8	327.0500; 301.0347; 300.0268; 285.0402; 284.0323; 271.0252; 256.0368; 255.0291; 229.0484; 227.0351; 178.9955; 151.0028	$C_{21}H_{20}O_{11}$	Kaempferol-3-O-glucoside	[M-H]⁻	0.799579	(Farag et al., 2013); (Amaral et al., 2009)
**1399**	57.71	515.1195	515.1189	-1.16	354.6903; 354.6140; 354.3911; 354.1977; 353.0874; 335.0756; 317.0658; 299.0571; 255.0652; 203.0341; 191.0556; 179.0344; 173.8701; 173.8218; 173.0448; 161.0231; 155.0346; 137.0240; 135.0447; 134.0365; 111.0440	$C_{25}H_{24}O_{12}$	Isochlorogenic acid C	[M−H]⁻	0.979344	(Malarz et al., 2025)
**618**	28.69	449.10894	449.1084	-1.19	287.0543; 269.0457; 259.0602	$C_{21}H_{22}O_{11}$	Hovetrichoside C	[M−H]⁻	0.849498	(Carvalho et al., 2026)
**537**	22.6	419.1195	419.119	-1.19	373.1135; 193.0503; 149.0600	$C_{16}H_{22}O_{10}$	Geniposidic acid	[M+FA]⁻	0.802606	(Zhou et al., 2010)

**513**	21.94	353.08781	353.0873	-1.43	191.0550; 179.0345; 173.0448; 135.0444	$C_{16}H_{18}O_9$	Cryptochlorogenic acid	$[M-H]^-$	0.960024	(Chen et al., 2011); (Xu et al., 2023)
**449**	16.77	353.08781	353.0873	-1.43	192.3275; 192.0088; 191.8638; 191.5599; 191.1042; 191.0555; 179.0346; 173.0448; 161.0242; 127.0397; 111.0451	$C_{16}H_{18}O_9$	Chlorogenic acid	$[M-H]^-$	0.969451	(Chen et al., 2011); (X. Zhang et al., 2022)
**287**	8.94	493.15628	493.1557	-1.17	447.1513; 293.0875; 233.0663	$C_{19}H_{28}O_{12}$	2-(3,4-Dimethoxyphenoxy)-6-[(3,4,5-trihydroxyoxan-2-yl)oxymethyl]oxane-3,4,5-triol	$[M+FA]^-$	0.873391	24150640
**781**	34.71	535.16684	535.1662	-1.2	489.1607; 195.0660; 151.0780; 149.0448; 125.0238	$C_{21}H_{30}O_{13}$	6'-O-beta-D-	$[M+FA]^-$	0.909008	10696407

							Apiofuranosylswerosid e			
**1311**	52.9 3	567.2083 2	567.20 8	-0.55	521.2067; 503.1904; 491.1900	$C_{26}H_{34}O_{11}$	3-[[2,3-Dihydro-2-(4-hydroxy-3-methoxyphenyl)-3-(hydroxymethyl)-7-methoxybenzofuran]-5-yl]propyl beta-D-glucopyranoside	[M+FA]⁻	0.986607	25225302
**1246**	48.3 9	769.2196 7	769.21 95	-0.22	315.0500; 314.0414; 300.0268; 299.0223	$C_{34}H_{42}O_{20}$	3-[(2S,3R,4S,5S,6R)-4,5-	[M−H]⁻	0.929852	(da Silva Pontes et al., 2024)

dihydroxy-
3-
[(2R,3R,4R
,5R,6S)-
3,4,5-
trihydroxy-
6-
methyloxan
-2-yl]oxy-
6-
[[(2R,3R,4
R,5R,6S)-
3,4,5-
trihydroxy-
6-
methyloxan
-2-
yl]oxymeth
yl]oxan-2-
yl]oxy-5,7-

							dihydroxy-2-(4-hydroxyphenyl)-6-methoxychromen-4-one			
**1006**	41.66	755.20402	755.2037	-0.42	711.2180; 645.2031; 619.1871; 591.1344; 573.1251; 547.1469; 489.1034; 465.1040; 445.1156; 409.0533; 373.0574; 355.0437; 343.0459; 325.0349; 300.0271; 281.0438; 271.0242; 255.0296; 243.0297; 227.0355; 215.0340; 178.9981; 169.0143; 163.0407; 151.0034	$C_{33}H_{40}O_{20}$	Manghaslin	$[M-H]^-$	0.951052	11498684
**1336**	53.39	515.11950	515.1189	-1.15	353.0860; 191.0552; 179.0345; 135.0449	$C_{25}H_{24}O_{12}$	1,3-Dicaffeoyl quinic acid	$[M-H]^-$	0.946716	(L. Zhang et al., 2026)

**559**	23.29	523.16684	523.1664	-0.85	447.1605; 293.0873; 233.0655; 125.0244	$C_{20}H_{30}O_{13}$	(2R,3S,4S,5R,6S)-2-[[(2S,3R,4R)-3,4-Dihydroxy-4-(hydroxymethyl)oxolan-2-yl]oxymethyl]-6-(3,4,5-trimethoxyphenoxy)oxane-3,4,5-triol	[M+FA]⁻	0.847362	23928091
**1884**	66.87	793.43798	793.4381	0.15	754.2486; 753.9469; 752.9217; 752.3043; 751.9738; 751.3442; 751.0522; 749.4484; 590.5026; 590.1800; 589.6234; 589.3746;	$C_{42}H_{66}O_{14}$	Rubbelliside A	[M−H]⁻	0.844328	23955890

	589.0748; 588.8033; 588.2871;<br>587.3950; 569.3883; 441.3363;<br>161.0446; 113.0234; 101.0240	

References

Åkesson, C., Lindgren, H., Pero, R. W., Leanderson, T., & Ivars, F. (2005). Quinic acid is a biologically active component of the Uncaria tomentosa extract C-Med 100®. *International Immunopharmacology*, *5*(1), 219–229. https://doi.org/10.1016/j.intimp.2004.09.028

Amaral, S., Mira, L., Nogueira, J. M. F., Silva, A. P. da, & Helena Florêncio, M. (2009). Plant extracts with anti-inflammatory properties—A new approach for characterization of their bioactive compounds and establishment of structure–antioxidant activity relationships. *Bioorganic & Medicinal Chemistry*, *17*(5), 1876–1883. https://doi.org/10.1016/j.bmc.2009.01.045

Antonio, A. da S., Oliveira, D. S., Cardoso dos Santos, G. R., Pereira, H. M. G., Wiedemann, L. S. M., & Veiga-Junior, V. F. da. (2021). UHPLC-HRMS/MS on untargeted metabolomics: a case study with *Copaifera* (Fabaceae). *RSC Advances*, *11*(40), 25096–25103. https://doi.org/10.1039/D1RA03163E

Arado, G. M., Amatto, P. de P. G., Marins, M., Rizzi, E. S., França, S. de C., Coppede, J. da S., Carmona, F., & Pereira, A. M. S. (2024). Anti-inflammatory and/or immunomodulatory activities of Uncaria tomentosa (cat's claw) extracts: A systematic review and meta-analysis of in vivo studies. *Frontiers in Pharmacology*, *15*. https://doi.org/10.3389/fphar.2024.1378408

Barboza, R. S., Mazzei, J. L., Valente, L. M. M., & Siani, A. C. (2015). Optimized Kaempferitrin Isolation from *Uncaria guianensis* Leaves by Solid-Phase Extraction. *Journal of Liquid Chromatography & Related Technologies*, *38*(4), 532–542. https://doi.org/10.1080/10826076.2014.917666

Batiha, G. E.-S., Magdy Beshbishy, A., Wasef, L., Elewa, Y. H. A., Abd El-Hack, M. E., Taha, A. E., Al-Sagheer, A. A., Devkota, H. P., & Tufarelli, V. (2020). Uncaria tomentosa (Willd. ex Schult.) DC.: A Review on Chemical Constituents and Biological Activities. *Applied Sciences*, *10*(8), 2668. https://doi.org/10.3390/app10082668

Blanck, J. J., Huebner, T. M., Rolls, A. M., Cornell, J. S., & Hwang, C. S. (2022). Comprehensive Review of the Components in Cat's Claw (Uncaria tomentosa) and Their Antibacterial Activity. *AppliedChem*, *2*(1), 1–29. https://doi.org/10.3390/appliedchem2010001

Carvalho, A. R. V., Reis, J. D. E., Ferraz, A. C., da Silva Menegatto, M. B., da C. Lopes, J., Pamplona, S. das G. S. R., da Silva, M. N., de Brito Magalhães, C. L., Gomes, P. W. P., & Yoshioka e Silva, C. Y. (2026). Unveiling the Molecular Profile and Antiviral

Potential of *Costus arabicus* L. Against Zika Virus. *Chemistry & Biodiversity*, *23*(2). https://doi.org/10.1002/cbdv.202503147

Chen, X., Lou, Z., Zhang, H., Tan, G., Liu, Z., Li, W., Zhu, Z., & Chai, Y. (2011). Identification of multiple components in *Guanxinning* injection using hydrophilic interaction liquid chromatography/time-of-flight mass spectrometry and reversed-phase liquid chromatography/time-of-flight mass spectrometry. *Rapid Communications in Mass Spectrometry*, *25*(11), 1661–1674. https://doi.org/10.1002/rcm.5003

Cunha, A. G., Alves Filho, E. G., Silva, L. M. A., Ribeiro, P. R. V., Rodrigues, T. H. S., Brito, E. S. de, & Miranda, M. R. A. de. (2020). Chemical composition of thermally processed coconut water evaluated by GC–MS, UPLC-HRMS, and NMR. *Food Chemistry*, *324*, 126874. https://doi.org/10.1016/j.foodchem.2020.126874

da Paixão, D., Barboza, R., Valente, L., Souza, M., Siani, A., Pereira, R., Gallo, B., & Berrueta, L. (2021). Polyphenol Profile and Quantitative Assessment of the Flavonoid Kaempferitrin in Wild and Cultivated Brazilian Amazonian Uncaria guianensis (Rubiaceae). *Journal of the Brazilian Chemical Society*. https://doi.org/10.21577/0103-5053.20210064

da Silva Pontes, A. L., Monteiro Leal, C., Pereira Lucas, M., Caamaño Muiño da Silva, G., Braga Alves Peixoto, J. V., Barbosa Succar, J., Ribeiro Flores, V., Neves Direito, I. C., Ribeiro da Silva, A. J., de Oliveira Chaves, F., & Eccard Fingolo, C. (2024). Dereplication Tools for *Rhizophora mangle* Extracts from Different Mangrove Areas and their Potential Against *Staphylococcus aureus*. *Chemistry & Biodiversity*, *21*(7). https://doi.org/10.1002/cbdv.202400687

Dreifuss, A. A., Bastos-Pereira, A. L., Fabossi, I. A., Lívero, F. A. dos R., Stolf, A. M., Alves de Souza, C. E., Gomes, L. de O., Constantin, R. P., Furman, A. E. F., Strapasson, R. L. B., Teixeira, S., Zampronio, A. R., Muscará, M. N., Stefanello, M. E. A., & Acco, A. (2013). Uncaria tomentosa Exerts Extensive Anti-Neoplastic Effects against the Walker-256 Tumour by Modulating Oxidative Stress and Not by Alkaloid Activity. *PLoS ONE*, *8*(2), e54618. https://doi.org/10.1371/journal.pone.0054618

Dührkop, K., Nothias, L.-F., Fleischauer, M., Reher, R., Ludwig, M., Hoffmann, M. A., Petras, D., Gerwick, W. H., Rousu, J., Dorrestein, P. C., & Böcker, S. (2021). Systematic classification of unknown metabolites using high-resolution fragmentation mass spectra. *Nature Biotechnology*, *39*(4), 462–471. https://doi.org/10.1038/s41587-020-0740-8

Farag, M. A., Weigend, M., Luebert, F., Brokamp, G., & Wessjohann, L. A. (2013). Phytochemical, phylogenetic, and anti-inflammatory evaluation of 43 Urtica accessions

(stinging nettle) based on UPLC–Q-TOF-MS metabolomic profiles. *Phytochemistry*, *96*, 170–183. https://doi.org/10.1016/j.phytochem.2013.09.016

Fawaid, A., & Mahfudh, N. (2025). Chemical Compound Profile of Bajakah Kalalawit (Uncaria gambir Roxb) Stem Extract Using Liquid Chromatography High-Resolution Mass Spectrometry. *Borneo Journal of Pharmacy*, *8*(1), 80–94. https://doi.org/10.33084/bjop.v8i1.7942

Ferreira, B. R., Campos, M. F., Rodrigues, S. B. F., Lima, A. B., Mendonça, S. C., Vilanova, C. M., Coutinho, D. F., Allonso, D., Amaral, F. M. M., & Leitão, S. G. (2026). From Traditional Knowledge to SARS-CoV-2 Entry Inhibitor Metabolites: Ethnopharmacological Investigation of Uncaria tomentosa (Willd. ex Schult.) DC. *Plants*, *15*(13), 1998. https://doi.org/10.3390/plants15131998

Kachlicki, P., Einhorn, J., Muth, D., Kerhoas, L., & Stobiecki, M. (2008). Evaluation of glycosylation and malonylation patterns in flavonoid glycosides during LC/MS/MS metabolite profiling. *Journal of Mass Spectrometry*, *43*(5), 572–586. https://doi.org/10.1002/jms.1344

Kodikara, C., Netticadan, T., Bandara, N., Wijekoon, C., & Sura, S. (2024). A new UHPLC-HRMS metabolomics approach for the rapid and comprehensive analysis of phenolic compounds in blueberry, raspberry, blackberry, cranberry and cherry fruits. *Food Chemistry*, *445*, 138778. https://doi.org/10.1016/j.foodchem.2024.138778

Li, Z., Huang, Q., Zheng, Y., Zhang, Y., Liu, B., Shi, W., & Zeng, Z. (2023). Kaempferitrin: A Flavonoid Marker to Distinguish Camellia oleifera Honey. *Nutrients*, *15*(2), 435. https://doi.org/10.3390/nu15020435

Lopes, A. A., Coppede, J. da S., Amatto, P. de P. G., Aragon, D. C., França, S. de C., Carmona, F., & Pereira, A. M. S. (2025). Cytotoxic effect of different Uncaria tomentosa (cat's claw) extracts, fractions on normal and cancer cells: a systematic review. *Frontiers in Pharmacology*, *16*. https://doi.org/10.3389/fphar.2025.1584840

Malarz, J., Jantas, D., Jakubowska, K., Bugno, R., Kiss, A. K., & Stojakowska, A. (2025). New Terpenoids and Polyphenolic Profile of Carpesium cernuum L. of European Origin. *Molecules*, *30*(12), 2506. https://doi.org/10.3390/molecules30122506

Navarro-Hoyos, M., Lebrón-Aguilar, R., Quintanilla-López, J., Cueva, C., Hevia, D., Quesada, S., Azofeifa, G., Moreno-Arribas, M., Monagas, M., & Bartolomé, B. (2017). Proanthocyanidin Characterization and Bioactivity of Extracts from Different Parts of Uncaria tomentosa L. (Cat's Claw). *Antioxidants*, *6*(1), 12. https://doi.org/10.3390/antiox6010012

Shiyan, S., Pratiwi, G., Handayani, S., Wibowo, J. T., Solihah, I., Hermansyah, H., Novitasari, L., Marsya, A. A., Fikria, N. A., Susilawati, S., & Indrasetiawan, P. (2025). In silico approach of gambier extract for Diabetes Mellitus and multivariate optimization of ultrasound-assisted extraction rich polyphenol using full factorial design. *Journal of Research in Pharmacy*, *29*(1), 190–209. https://doi.org/10.12991/jrespharm.1634460

Sumner, L. W., Amberg, A., Barrett, D., Beale, M. H., Beger, R., Daykin, C. A., Fan, T. W.-M., Fiehn, O., Goodacre, R., Griffin, J. L., Hankemeier, T., Hardy, N., Harnly, J., Higashi, R., Kopka, J., Lane, A. N., Lindon, J. C., Marriott, P., Nicholls, A. W., … Viant, M. R. (2007). Proposed minimum reporting standards for chemical analysis. *Metabolomics*, *3*(3), 211–221. https://doi.org/10.1007/s11306-007-0082-2

Sun, X., Shang, Z., Zhang, Y., Qiu, J., & Tan, X. (2024). Comprehensive Investigation of Sweroside Metabolism in Rats Using a De Novo Strategy Based on Ultra-High-Performance Liquid Chromatography/Quadrupole-Exactive Mass Spectrometry. *Journal of Separation Science*, *47*(23). https://doi.org/10.1002/jssc.70048

Wang, H., He, X., Tong, S., Dai, X., Wu, Q., Hu, J., Dong, C., Wang, J., Lei, C., Feng, J., Li, W., Zhang, L., Huang, Q., & Pu, X. (2025). Functional characterization of loganic acid O-methyltransferase and secologanin synthase from Uncaria rhynchophylla and two-step biosynthesis of secologanin in tobacco. *International Journal of Biological Macromolecules*, *333*, 148806. https://doi.org/10.1016/j.ijbiomac.2025.148806

Xu, X., Wen, J., Wang, S., Hao, J., Du, K., Fang, S., He, J., Li, J., & Chang, Y. (2023). Simultaneous extraction and determination of alkaloids and organic acids in Uncariae Ramulas Cum Unicis by vortex-assisted matrix solid phase dispersion extraction coupled with UHPLC-MS/MS. *Frontiers in Chemistry*, *11*. https://doi.org/10.3389/fchem.2023.1100150

Zhang, L., Dai, W., Tian, Y., Luo, P., Wang, S., Zheng, J., Sun, P., & Wang, Q. (2026). Optimized normalized collision energy and characteristic ion fragments for LC-MS/MS identification of caffeoylquinic acid isomers using cosine similarity. *Talanta*, *308*, 129848. https://doi.org/10.1016/j.talanta.2026.129848

Zhang, X., Wang, S., Shu, L., Zhao, S., Yan, X., Jia, G., Zhang, Y., Zhang, W., Qian, W., Yang, B., & Li, Y. (2022). Rapid screening of hepatotoxic components in Uncariae Ramulus Cum Uncis based on "component-target-pathway" network. *Journal of Pharmaceutical and Biomedical Analysis*, *219*, 114968. https://doi.org/10.1016/j.jpba.2022.114968

Zhou, T., Liu, H., Wen, J., Fan, G., Chai, Y., & Wu, Y. (2010). Fragmentation study of iridoid glycosides including epimers by liquid chromatography-diode array detection/electrospray ionization mass spectrometry and its application in metabolic fingerprint analysis of *Gardenia jasminoides* Ellis. *Rapid Communications in Mass Spectrometry*, *24*(17), 2520–2528. https://doi.org/10.1002/rcm.4643
